# Protocol of a mixed-methods evaluation of Perfect Fit: A personalized mHealth intervention with a virtual coach to promote smoking cessation and physical activity in adults

**DOI:** 10.1177/20552076241300020

**Published:** 2024-12-05

**Authors:** Milon H. M. van Vliet, Anke Versluis, Niels H. Chavannes, Bouke L. Scheltinga, Nele Albers, Kristell M. Penfornis, Walter Baccinelli, Eline Meijer, on behalf of the Perfect Fit consortium

**Affiliations:** 1 Department of Public Health and Primary Care, 4501 Leiden University Medical Center, Leiden, The Netherlands; 2 National eHealth Living Lab, 4501 Leiden University Medical Center, Leiden, The Netherlands; 3 Biomedical Signals and Systems, 3230 University of Twente, Enschede, The Netherlands; 4 Roessingh Research and Development, Enschede, The Netherlands; 5 Intelligent Systems, 2860 Delft University of Technology, Delft, The Netherlands; 6 Unit Health-, Medical and Neuropsychology, 4496 Leiden University, Leiden, The Netherlands; 7 443478 Netherlands eScience Center, Amsterdam, The Netherlands

**Keywords:** mHealth, mobile health, virtual coach, coaching, activity trackers, smoking, low physical activity, mixed-methods evaluation

## Abstract

**Objective:**

Adopting healthy behavior is vital for preventing chronic diseases. Mobile health (mHealth) interventions utilizing virtual coaches (i.e., artificial intelligence conversational agents) can offer scalable and cost-effective solutions. Additionally, targeting multiple unhealthy behaviors, like low physical activity and smoking, simultaneously seems beneficial. We developed Perfect Fit, an mHealth intervention with a virtual coach providing personalized feedback to simultaneously promote smoking cessation and physical activity. Through innovative methods (e.g., sensor technology) and iterative development involving end-users, we strive to overcome challenges encountered by mHealth interventions, such as shortage of evidence-based interventions and insufficient personalization. This paper outlines the content of Perfect Fit and the protocol for evaluating its feasibility, acceptability, and preliminary effectiveness, the role of participant characteristics, and the study's feasibility.

**Methods:**

A single-arm, mixed-method, real-world evaluation study will be conducted in the Netherlands. We aim to recruit 100 adult daily smokers intending to quit within 6 weeks. The personalized intervention will last approximately 16 weeks. Primary outcomes include Perfect Fit's feasibility and acceptability. Secondary outcomes are preliminary effectiveness and study feasibility, and we will measure participant characteristics. Quantitative data will be collected through questionnaires administered at baseline, post-intervention and 2, 6, and 12 months post-intervention. Qualitative data will be gathered via semi-structured interviews post-intervention. Data analysis will involve descriptive analyses, generalized linear mixed models (quantitative) and the Framework Approach (qualitative), integrating quantitative and qualitative data during interpretation.

**Conclusions:**

This study will provide novel insight into the potential of interventions like Perfect Fit, as a multiple health behavior change strategy. Findings will inform further intervention development and help identify methods to foster feasibility and acceptability. Successful mHealth interventions with virtual coaches will prevent chronic diseases and promote public health.

## Introduction

Unhealthy behaviors like low physical activity (PA) and tobacco smoking are significant preventable risk factors for chronic diseases, such as cardiovascular diseases and various types of cancer, and premature death.^1^ Recent studies indicate that globally, smoking accounts for 20.2% of male deaths and 5.8% of female deaths,^2^ while physical inactivity accounts for 7.2% of deaths.^3^ Hence, reducing tobacco use^4^ and increasing PA^5^ are among the most important strategies to reduce chronic disease mortality.^1^ Although adopting healthy behavior is a well-known strategy to prevent chronic diseases, approximately 22% of the world's adult population still smoke tobacco, and over 25% are insufficiently active.^5,6^ Consequently, the prevalence of chronic diseases is still rising, and chronic diseases currently lead to 17 million premature deaths each year globally.^1,7^ Traditional face-to-face interventions have proven to be successful in health promotion,^7–9^ but often face challenges like low user engagement,^10^ logistic and time constraints, cost-effectiveness problems^9^ and low accessibility.^8,11^ Additionally, the global shortage of healthcare personnel severely restricts the capacity for face-to-face interventions.^12^ Hence, there is a discrepancy between the high global burden of chronic diseases and the available support to help people adopt and maintain healthy behavior.^7,11,12^ This is worrisome since health behavior change is often more successful when receiving support.^8,13^ Therefore, optimizing the delivery of effective health behavior change interventions is vital.

eHealth has the potential to increase accessibility, reach, adaptability^11,12^ and cost-effectiveness^11,14^ of health behavior change interventions. eHealth can be defined as health services and information delivered or enhanced through the Internet and related technologies.^15^ eHealth interventions have demonstrated effectiveness comparable to traditional face-to-face interventions^11^ and can be deployed with lower, or no, involvement of health professionals, making them an interesting approach to improve healthcare efficiency.^12,16^ Specifically, health services and information supported by mobile devices, known as mHealth interventions, are increasingly being used due to their potential to exchange health-related information and provide support in a fast, scalable and cost-effective manner. Given the rapid increase in the number of people with mobile phones, also in low- and middle-income countries, mHealth interventions could potentially also reach individuals who have limited access to healthcare.^17^

Literature shows that adherence to online health behavior change interventions can be enhanced when combined with human support, as it can create social support, feedback and accountability.^11,18^ This support can also be in the form of online support from a virtual coach, provided it employs strategies to enhance the user's relationship with the virtual coach.^11^ Virtual coaches are artificial intelligence conversational agents that can mimic human conversations using text, spoken language or both.^10,14,19^ Combining mHealth interventions with the support of a virtual coach is suggested to increase user engagement and effectiveness while still having the advantages of mobile-delivered interventions.^11^ Two previous reviews have highlighted the potential effectiveness of virtual coach interventions in promoting PA^14^ and smoking cessation.^10^ Furthermore, the review of He et al.^10^ provides initial evidence supporting positive user experiences and high engagement. These promising findings, coupled with the capability of virtual coaches to be always available for multiple individuals simultaneously,^10,14^ make virtual coach interventions an interesting health promotion strategy.

Many mHealth behavior change interventions which employ virtual coaches target a single health behavior.^10,14^ Research suggests, however, that targeting multiple health behaviors simultaneously can be beneficial, as unhealthy behaviors, such as low PA and smoking, often co-occur.^7,20^ Additionally, successful change in one health behavior can enhance self-efficacy and motivation for changing another.^20^ Studies have highlighted the benefits of addressing both low PA and smoking in a single intervention. For example, an exercise intervention in smokers has demonstrated that engaging in PA decreases negative affect and nicotine cravings, thereby increasing the chances of successful smoking cessation.^21^ Conversely, an observational study showed that quitting smoking can improve fitness, thereby facilitating PA.^22^ As PA promotion and smoking cessation also reduce the risks for many chronic diseases, simultaneously targeting these health behaviors in a multi behavior change intervention may be efficient.^22–24^ Moreover, research suggests that aligning behavioral goals with a higher-order conceptual goal can facilitate goal attainment. For instance, aspiring to become a healthy person, can aid in achieving specific behavioral goals such as increasing health-promoting behaviors (e.g., PA) and discontinuing health-compromising behaviors (e.g., smoking).^25^ Hence, an intervention providing guidance on common behavior change principles, along with specific strategies for applying these principles to both PA and smoking cessation, could be an efficient approach for individuals facing multiple health behavior risks.^26,27^

The interdisciplinary consortium ‘Perfect Fit’ has developed an mHealth intervention with a virtual coach providing personalized, real-time, text-based and visual feedback to simultaneously promote smoking cessation and PA in adults. Despite the great potential of mHealth interventions with a virtual coach, previous reviews have also highlighted challenges such as lack of evidence- and theory-based interventions,^10,12^ insufficient tailoring and personalization,^10,14^ the need for users to have a certain level of (health) literacy and digital skills^12,17^ and low long-term user engagement.^10^ In the development of Perfect Fit, we have aimed to address these challenges.

First, Perfect Fit integrates behavior change taxonomies,^e.g.,^^28^ the Relapse Prevention Model^29^ and theories of identity change (e.g., to become a physically active person or non-smoker),^30–32^ components from existing interventions (e.g., StopCoach)^33^ and findings from previous (empirical) studies by our research group.^e.g.,^^34–39^ Theoretical foundations increase the likelihood of interventions being successful and help to understand potential mechanisms of behavior change.^10,12^ Second, Perfect Fit is personalized and tailored in various ways. For instance, the virtual coach provides real-time and personalized feedback using sensor technology (i.e., smartwatch data). Moreover, the coach is both system- and user-initiated and allows a combination of fixed and free-text answers. Users can flexibly adjust intervention aspects such as content, timing, and duration based on their needs. This personalization not only makes the coach more human-like but can also enhance users’ sense of ownership and autonomy, potentially increasing effectiveness and user engagement.^10–12^ Third, Perfect Fit is designed to maximize accessibility, especially for users with lower (health) literacy or limited digital skills. To achieve this, end-users were involved in the early stages of development and prototype testing with iterative feedback was employed. Furthermore, experts, including specialists in tailoring research to populations with lower socio-economic position (SEP), were consulted. This collaborative process has led to the inclusion of intervention components such as animated educational videos and the use of easy-to-understand language (i.e., B1-level Dutch), ensuring inclusivity and user-friendliness.^12,17^ Fourth, by incorporating the aforementioned strategies and innovative techniques, we aim to increase long-term engagement. Moreover, relational aspects, such as empathy and the use of emoticons, are integrated into the virtual coach to strengthen the user-coach relationship, potentially increasing long-term engagement.^10,11,40^ A detailed description of the development of Perfect Fit and lessons learned is reported elsewhere.^41^

In this paper, we describe the content of Perfect Fit and outline the study protocol for evaluating the feasibility, acceptability and preliminary effectiveness of the intervention and the virtual coach using a single-arm, concurrent mixed-methods design. Additionally, associations between individual participant characteristics, feasibility, acceptability and preliminary effectiveness parameters will be examined. Finally, the feasibility of the research study will be evaluated. Reporting on the feasibility and acceptability of mHealth interventions is currently limited.^14,17^ Although evidence of effectiveness is a basic requirement for implementation, effectiveness and user experience are intricately related.^10,42^ With this study, we aim to gain more insight into whether Perfect Fit can overcome the challenges that mHealth interventions typically face. Developing feasible and effective mHealth interventions for promoting PA and smoking cessation is crucial to alleviate the burden on healthcare systems and professionals, reduce the impact of chronic conditions and ultimately promote public health.

## Methods

### Design

A single-arm, concurrent mixed-method, real-world evaluation study with a pretest-posttest design will be conducted in the Netherlands. The intervention duration is personalized, but is expected to be on average 16 weeks. The total study duration per participant is 16 months (i.e., 68 weeks). The quantitative part consists of self-report questionnaires on feasibility, acceptability and preliminary effects of Perfect Fit, usage data, objective level of PA (measured with a smartwatch), and research procedures monitored with a screening and inclusion logfile. The questionnaires will be administered at baseline (week 0 [T0]), post-intervention (week 16 [T1]), and at three follow-up time points (2 months [T2], 6 months [T3] and 12 months [T4] after the expected end of the intervention). The qualitative part consists of semi-structured individual interviews, conducted post-intervention, and will complement the quantitative results. These interviews will focus on preliminary effectiveness, feasibility (e.g., usage), acceptability (e.g., relationship with virtual coach), study feasibility, and requirements for implementation.

The study was approved by the Medical Research Ethics Committee Leiden The Hague Delft (N23.045 METC-LDD) on July 17, 2023, and was registered at ClinicalTrials.gov (NCT06095999) in October 2023. The study protocol follows the SPIRIT 2013 guidelines,^43^ the SPIRIT-outcomes 2022 extension^44^ and the CONSORT- EHEALTH checklist (V1.6.1).^45^

### Participants

To be eligible to participate participants must meet the following inclusion criteria (based on self-report): 1) age ≥ 18 years, 2) current daily smoker (cigarette, cigar, or rolling tobacco), 3) having the intention to quit smoking between now and 6 weeks, 4) being able to walk pain free, 5) being able to understand and read Dutch (at least B1 level), and 6) having a smartphone with an Internet connection. To examine Perfect Fit in high-risk individuals, at least 50% of participants should have a ≥ 10% risk of cardiovascular diseases according to the Dutch general practice guideline for cardiovascular risk management.^46^ Specifically, this will be estimated based on being female and 55 + years old or male and 50 + years old. Finally, at least 75% of participants should live in the region of Leiden (i.e., the region between Leiden, Amsterdam and Utrecht in the Netherlands).

Exclusion criteria are: 1) Being involved in smoking cessation treatment at the start of the intervention, 2) having undergone major lower extremity surgery in the past year (to prevent giving PA advice that contradicts medical guidance), 3) taking antipsychotics or having a serious psychiatric illness (e.g., schizophrenia/psychosis, bipolar disorder, major depression), and 4) being pregnant. Participants are not excluded based on PA levels, as even active individuals can benefit from increasing activity, and daily smokers can still significantly benefit from quitting, regardless of their activity level.

### Sample size

Previously conducted comparable (mixed-methods) single-arm studies, investigating the feasibility, acceptability and/or preliminary effectiveness of an mHealth or eHealth intervention, reported sample sizes between 30 and 56.^47–50^ This aligns with recommendations for sample sizes of usability/acceptability studies.^51^ It is known that mHealth intervention studies often have high attrition rates^52^ and previously conducted comparable studies indeed report attrition rates between 7% and 30%.^47–50^ Therefore, we aim to include 100 participants in our study, allowing for a dropout rate of 44%.

For the individual semi-structured interviews with participants, we aim to recruit a heterogeneous subsample considering differences in individual characteristics (e.g., gender, age), (health) literacy, adherence to Perfect Fit, and success of health behavior change. We expect to include around 10–15 participants to achieve data saturation.^53^

### Setting and recruitment

The study will take place in the Netherlands and will primarily be conducted online (e.g., introduction meeting via video calling, online self-report questionnaires). Participants will be recruited through a combination of online and offline strategies. Online recruitment will involve social media posts and advertisements (Facebook and Linkedin), news articles on the Perfect Fit website and newsletters of affiliated organizations (e.g., Trimbos-institute and healthcare insurer Zorg & Zekerheid). Offline recruitment will be facilitated through a news item in a local newspaper and by enlisting the help of Perfect Fit consortium partners and the Perfect Fit advisory board, who will distribute recruitment flyers within their network. Additionally, news items will be featured in magazines for healthcare professionals, asking them to inform individuals about the study. Finally, participants from a prior project study who had expressed interest in follow-up research will be invited to participate.

Recruitment started in August 2023 and ended when the intended sample size was reached, on February 12, 2024. Data collection (i.e., first inclusion) started in September 2023 and is expected to end (i.e., final follow-up questionnaire completed) in May 2025.

### Intervention

Perfect Fit is the result of interdisciplinary collaboration and is an evidence-based, personalized program in which users receive support from a virtual coach to help them quit smoking and become more physically active. The development, intervention content, design and implementation of Perfect Fit, as well as lessons learned, are described elsewhere.^41^

Perfect Fit spans approximately 16 weeks, guiding participants through three phases with the virtual coach, ‘Sam’. In the preparation phase, participants engage in activities to prepare for smoking cessation and increasing PA, supported by the coach through chat conversations and informative animation videos on topics such as medication and nicotine replacement therapy, and goal setting. During the execution phase, participants focus on quitting smoking and increasing PA, supported by the coach through various components, such as activities to facilitate behavior change and weekly reflections. In the closing phase, participants engage in a final chat conversation to review their progress and results, and develop a relapse prevention plan. A comprehensive overview of Perfect Fit components and features, including their aims and the evidence supporting them, is provided in Appendix A. While some components are the same for all users, the personalized intervention allows users to choose optional components and decide whether to consult external links provided. Users are free to use additional PA or smoking cessation aids (e.g., medication, websites, booklets) and can adjust the timing of components to their preference.

#### The virtual coaching system

The Perfect Fit virtual coaching system runs on a cloud system and the user can interact with it through an existing smartphone app equipped with chat functionality (i.e., the NiceDay app used for remote therapy^a^), which is available for both Android and iOS systems. The coaching system allows both system- and user-initiated chat conversations, and a combination of constrained (i.e., selecting pre-programmed responses) and unconstrained (i.e., free-reponse format) user input. The virtual coach is represented by the face of an animated robot (i.e., non-embodied) named ‘Coach Sam’ and offers text-based support enriched with emoticons, images, graphs and links to educational videos and external sources (see Appendix B for screenshots). The coach utilizes artificial intelligence techniques and input from users (e.g., user states) and employs persuasive (e.g., goal-setting) and relational strategies (e.g., being empathic). Additionally, sensor data (i.e., number of steps) measured by the smartwatch is integrated into the personalized PA coaching provided by the coach. A description of the technical architecture of the open-source text-based coaching system, which integrates big-data science, sensor technology, and personalized real-time feedback, can be found elsewhere.^54^ Finally, to promote reusability and accessibility, the source code of the Perfect Fit system^b^ is publicly available.^55^

### Procedure

Interested individuals can register via the study website^c^ or by sending an e-mail to the researchers. Subsequently, these individuals receive a digital information letter and a link to the online screening questions (using secured software Castor EDC^®^) via e-mail. Eligible individuals are asked to sign the online informed consent form, which is a requirement for study participation. Participants will be informed that they can withdraw from the study at any time for any reason if they wish to do so.

**Figure 1.**
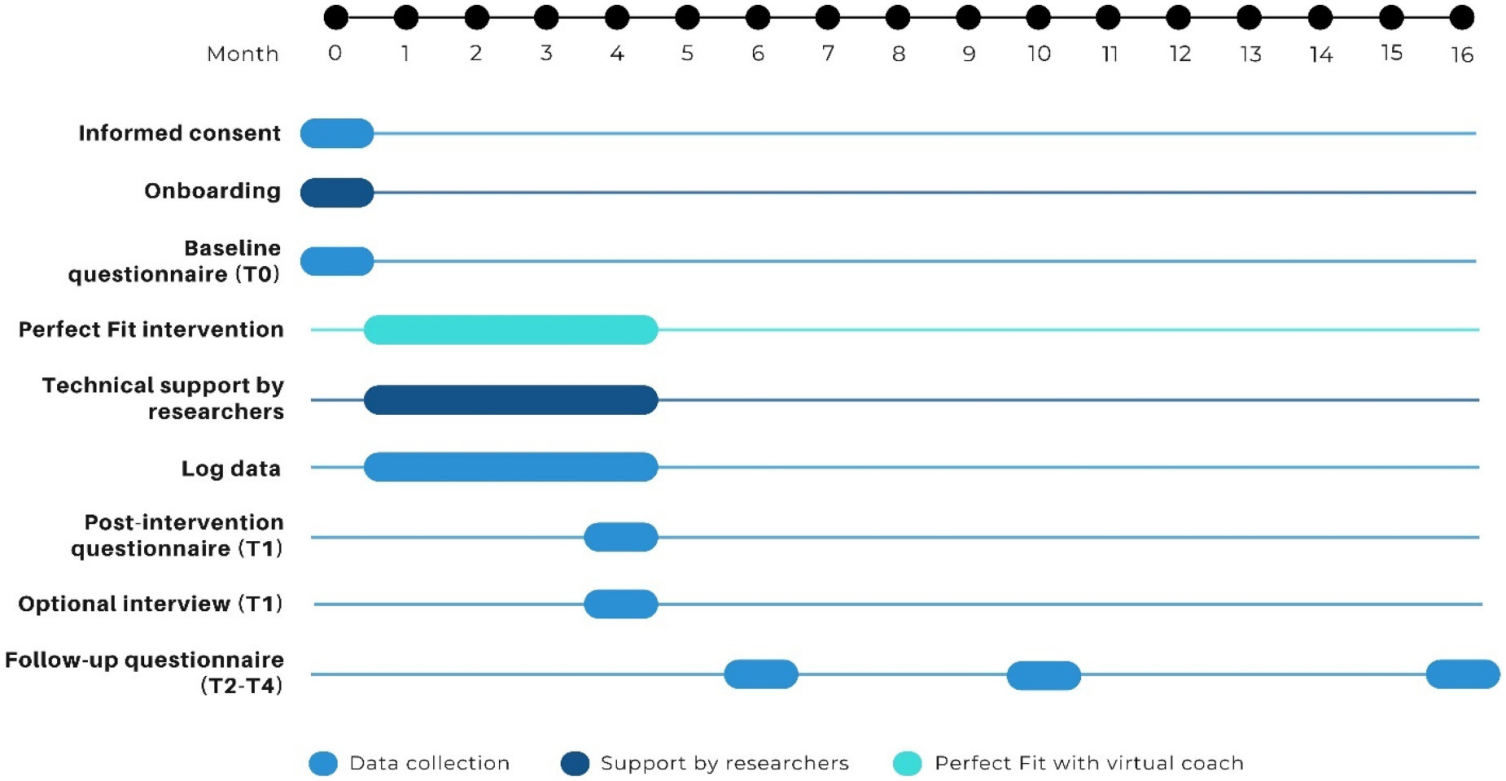
Participant timeline.

A timeline for participants is shown in Figure 1. After providing informed consent, a researcher will call the participant to explain the onboarding procedure and schedule an online onboarding meeting. Next, the participant will receive a smartwatch (Garmin Forerunner 55) plus information materials and instructions. The materials will include: 1) a video explaining the study, the intervention and the smartwatch (e.g., rationale, privacy, global overview of the intervention, how to use the smartwatch), 2) an installation booklet with instructions for downloading the necessary apps and connecting with the virtual coach, and 3) an information booklet with a summary of important instructions and information, including tips on how to communicate with the virtual coach. The information booklet can also be used as an exercise book, as it provides space for notes. The link to the video will be sent via e-mail, and the booklets and smartwatch will be sent via postal service. After receiving all the materials, the participant will have the onboarding meeting with a researcher via video call or telephone. During this meeting, the researcher will verify if the participant has successfully installed the apps, understands how to wear the smartwatch, and has connected with the virtual coach. Any remaining questions will be addressed during this meeting. The researchers will offer assistance to participants via e-mail or telephone when necessary (e.g., in case of technical problems). Some optional short activities and educational videos from the intervention will also be accessible through the study website should participants wish to revisit them post-intervention.

The link to the online baseline questionnaire (using software Castor EDC^®^) will be sent in the week of the onboarding meeting (T0), and the link to the post-intervention questionnaire will be sent 16 weeks later (i.e., expected end of the intervention, T1). Invitations for online follow-up questionnaires will be sent via e-mail 2 months (T2), 6 months (T3) and 12 months (T4) after the expected end of the intervention. The researchers will monitor questionnaire completion and send reminders via e-mail and phone calls as needed. Additionally, the Castor EDC^®^ feature requiring participants to answer every question will be used to prevent missed responses. As an incentive for completing the study, participants may keep the smartwatch, if they complete at least 80% of the questionnaires. No additional monetary compensation is provided for completing Perfect Fit and the questionnaires.

A subsample of the participants will be invited for a semi-structured individual interview via e-mail (see Appendix C for the interview protocol) at T1, but only if they provided their informed consent at the beginning of the study to be contacted for this purpose. The interviews will last 45–60 min and will be mainly conducted online, or via telephone or face-to-face if the participant strongly prefers this. The interviews will be conducted by two Medicine master's students trained in interviewing, of which one will take notes during the interview. Before the start of the interview, verbal informed consent from the participant will be obtained and audio-recorded. Participants will receive a gift voucher of 25 euros for the interview, along with the option to keep the smartwatch.

### Outcomes

An overview of the instruments used to assess the study outcomes at each time point is presented in Table 1. Primary outcomes are the intervention's feasibility, acceptability, and usability, and the acceptability and usability of the virtual coach. Secondary outcomes are preliminary effectiveness and study feasibility. A more detailed description of the different study outcomes can be found in Appendix D.

**Table 1.** Overview of outcomes, instruments, data collection methods and assessment time points.

	Method	Time points					
		T0	Int.	T1	T2	T3	T4
		*Wk 0*	*Wk 1–16*	*Wk 16*	*Wk 24*	*Wk 40*	*Wk 64*
**Primary outcomes**							
*Intervention's feasibility*							
Usage data on completed intervention components	L		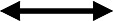				
Frequency contact coach (1 item)	Q			X			
*Intervention's acceptability*							
Satisfaction with the intervention (2 items)	Q			X			
*Intervention's usability*							
SUS (10 items)^56^	Q			X			
*Acceptability and usability coach*							
Acceptability and usability of the coach (7 items)^57,58^	Q			X			
Qualitative interview data on primary outcome measures	I			X			
**Secondary outcomes**							
*Preliminary effectiveness*							
Smoking abstinence (2–3 items)	Q			X	X	X	X
Level of PA: GSLTPAQ (3 items)^59,60^	Q	X		X	X	X	X
Non-smoker self-identity (7 items)	Q	X		X	X	X	X
PA self-identity: adapted EIQ (9 items)^61,62^	Q	X		X	X	X	X
Smoking abstinence self-efficacy (1 item)	Q	X		X	X	X	X
PA self-efficacy (1 item)	Q	X		X	X	X	X
Objective level of PA: number of steps (smartwatch)	S	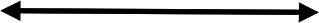					
*Study feasibility*							
Recruitment, response and consent rate^a^	R						
Recruitment strategies (1 item)	Q	X					
Users’ adherence^b^	R	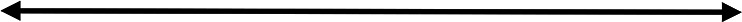					
Intervention completion (1 item)	Q			X	X	X	X
Qualitative interview data on secondary outcome measures	I			X			
**Other variables**							
Participant characteristics, including eHLQ (30 items)^63,64^ ^c^	Q	X					
Intention to quit smoking (1 item)	Q	X					
Smoking behavior and nicotine dependence: FTND (6 items)^65^	Q	X					
Intention to become more physically active (1 item)	Q	X					
Use of additional aids/support for smoking cessation (1 item)	Q			X	X	X	X
Use of additional aids/support for PA (1 item)	Q			X	X	X	X
Self-reported (re)lapses during intervention after quit date	L		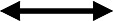				
Smoking e-cigarettes (1–3 items)	Q	X		X	X	X	X
Self-reported level of occupational PA (1 item)	Q	X		X	X	X	X

^a^ Recruitment, response and consent rate will be assessed from the start of the recruitment phase until the inclusion of the final participant.

^b^ Users’ adherence will be assessed from the moment informed consent is obtained until T4.

^c^ A license for the use of the eHealth Literacy Questionnaire was obtained from the copyright holders.

T0 = baseline; Int. = During intervention; T1 = Post-intervention; T2 = 2-month follow-up; T3 = 6-month follow-up; T4 = 12-month follow-up; Wk = Week.

L = log data of coaching system; Q = self-reported questionnaire; I = semi-structured individual interview; S = smartwatch; R = researchers using a participant screening and inclusion logfile.

SUS = System Usability Scale; PA = Physical activity; GSLTPAQ = Godin-Shephard Leisure-Time Physical Activity Questionnaire; EIQ = Exercise Identity Questionnaire; eHLQ = eHealth Literacy Questionnaire; FTND = Fagerström Test of Nicotine Dependence.

### Data analyses

Quantitative and qualitative data will be analysed independently and integrated at the point of interpretation.

#### Quantitative analyses

Descriptive analyses will be performed in SPSS (SPSS 25.0 statistics for Windows, IBM, Armonk, New York) and generalized linear mixed models in RStudio (RStudio Version 2023.12.1 + 402, PBC, Boston, MA, http://www.rstudio.com/). Outliers and assumptions will be checked before data analyses are conducted. A *p*-value of < .05 will be considered statistically significant.

Descriptive analyses (e.g., *M *± *SD*, N, percentages) will be conducted to summarize the participant characteristics (e.g., age, eHealth literacy) and the characteristics related to PA (e.g., intention to become more physically active) and smoking (e.g., physical nicotine dependence and intention to quit). Descriptive analyses will also be conducted to describe quantitative data concerning the interventions’ feasibility, acceptability and usability, the acceptability and usability of the virtual coach, and the study feasibility.

To examine whether potential changes over time in preliminary effectiveness outcomes are significant compared to baseline, we will use generalized linear mixed models for ordinal data (abstinence self-efficacy, PA self-efficacy, non-smoker self-identity, PA self-identity), continuous data (number of steps) and binary data (prolonged smoking abstinence and level of PA). For each outcome variable, a separate model will be run. The outcome variable will serve as the dependent variable, with time (i.e., T0-T4) as a fixed effect and subject as a random effect. This will allow us to examine whether there is a significant change over time in these preliminary effectiveness outcomes and to examine whether the change over time differs per participant. Generalized mixed models were chosen because they can handle missing values implicitly while retaining maximum power, and these analyses can deal with unequal time intervals between measurements.

We also intend to perform exploratory subgroup analyses, depending on the power for these analyses. This could provide insight into, for instance, the relation between SEP and the preliminary effectiveness of Perfect Fit. To do so, the individual characteristic, in this case, SEP, will be included as a covariate in the generalized mixed model (i.e., allowing us to identify whether SEP impacts change over time in preliminary effectiveness outcomes). Data will be pseudonymized, processed confidentially, and securely stored on an internal drive. Access to data will be restricted to study personnel. The data will be stored for 15 years.

#### Qualitative analyses

All individual semi-structured interviews will be audio-recorded, anonymized, and transcribed verbatim for subsequent analyses. Qualitative data analysis will be conducted following the principles of the Framework Approach^66,67^ and using software ATLAS.ti (ATLAS.ti Version 23.2.3.27778, 2023, ATLAS.ti Scientific Software Development GmbH, https://atlasti.com/). The Framework Approach is a structured and transparent qualitative analysis method that uses matrices to organize and analyze data by case (i.e., participant) and by code. It is chosen for its ease of use, particularly when multiple (quantitative) researchers are involved, and because it combines both inductive and deductive analysis.^66,67^ The first transcripts will be independently coded by the two master's students who conduct the interviews and MV, resulting in an initial coding scheme. Subsequently, the students will proceed to code the remaining transcripts and discuss any adjustments to the coding scheme with the author team. Data interpretation and theme generation will be conducted by MV, in collaboration with the author team. During data analyses, the interviews will be securely stored on an internal drive. Upon completion of data analysis, the audio records will be deleted.

## Discussion

This paper presents the novel mHealth intervention called Perfect Fit and outlines the protocol for a single-arm, concurrent mixed-method, real-world evaluation of Perfect Fit. Perfect Fit utilizes a virtual coach providing personalized, real-time, text-based, and visual feedback to promote smoking cessation and PA in adults. The main aim of this study is to examine the feasibility and acceptability of Perfect Fit. Additionally, the preliminary effectiveness of Perfect Fit, and associations between individual characteristics and parameters of feasibility, acceptability and/or preliminary effectiveness will be explored. Finally, the overall feasibility of the study will be evaluated. The combination of quantitative and qualitative methods will enable us to contextualize the quantitative data, leading to a deeper understanding of our findings.

Although mHealth interventions with virtual coaches are a promising health promotion strategy with many benefits (e.g., scalable and cost-effective),^14,17^ they also encounter challenges.^10,14^ With Perfect Fit, we aim to address challenges such as the scarcity of evidence- and theory-based interventions,^10,12^ insufficient tailoring and personalization,^10,14^ the need for users to have a certain level of (health) literacy and digital skills^12,17^ and low long-term user engagement.^10^ Through an iterative development process involving an interdisciplinary team and end-users and by making use of innovative techniques (e.g., sensor technology), tailoring and personalization, we strive to overcome common challenges faced by mHealth interventions. Moreover, to our knowledge, integrating both a health-compromising behavior, like smoking, and a health-promoting behavior, like PA, into a single mHealth intervention is novel.

Our primary aim is to investigate the feasibility and acceptability of Perfect Fit as a whole and the virtual coach itself. Considering the interplay between user characteristics and technology is essential for ensuring the long-term use and implementation of mHealth interventions.^16^ By gaining insights into user experiences and needs, including how users perceive the engagement with Perfect Fit and their relationship with the virtual coach, we aim to contribute valuable knowledge for the development of new mHealth interventions with long-term engagement. We also hope to gain first insights into criteria for future implementation strategies, such as determining whether mHealth interventions with virtual coaches are better suited as stand-alone or an add-on intervention (e.g., to pharmacological treatment), and for whom they are most effective. Furthermore, we investigate the feasibility of the study itself, aiming to provide insights that can inform the design of future studies investigating mHealth interventions. This is particularly relevant given the high attrition rates observed in these studies.^52^

Another aim is to investigate the preliminary effectiveness of Perfect Fit, in which we simultaneously target PA enhancement and smoking cessation. Investigating the preliminary effectiveness of Perfect Fit will provide valuable insights into the effectiveness of targeting multiple health behaviors simultaneously.^7,20^ Specifically, these findings will shed light on the effectiveness of integrating PA enhancement and smoking cessation within a single intervention. Additionally, in this study, PA levels and smoking abstinence will be monitored up to 12 months post-intervention. Health behavior change is a dynamic process, which is particularly evident in smoking cessation,^68^ and long-term behavior change can be difficult to achieve.^9,10^ Despite this, many studies on mHealth interventions for smoking cessation lack long follow-up periods.^10^ However, research shows that short-term smoking abstinence immediately following an intervention does not reliably predict long-term abstinence.^68,69^ By incorporating a 12-month follow-up period, we aim to gain insight into the potential of Perfect Fit to facilitate long-term health behavior change.

Exploratory subgroup analyses will also indicate whether the intervention is more feasible, acceptable or effective for certain populations (e.g., individuals with low eHealth literacy or SEP), guiding further development. mHealth interventions with virtual coaches have the potential to reach individuals who have limited access to healthcare, but may also unintentionally increase health disparities.^10,16^ Therefore, it is vital that mHealth interventions are developed and implemented in a way that meets the characteristics, needs and preferences of these individuals. With this study, we aim to gain insight into the extent to which Perfect Fit meets the needs of individuals with diverse socio-demographic characteristics.

This study also has some limitations. Although we aimed to develop Perfect Fit to be accessible for users with lower (health) literacy and digital skills (e.g., through the use of animation videos and B1-level Dutch), users still need to be able to read text-based chat messages, have basic digital skills, and have access to a smartphone to use Perfect Fit. As a result, the findings of this study may not represent the experiences of individuals who lack these skills or who do not have access to a smartphone. However, participants’ eHealth literacy will be measured at baseline to better understand for which users, in terms of eHealth literacy levels, Perfect Fit is suitable. Another limitation is that Perfect Fit will be investigated as a stand-alone intervention, as we want to assess the feasibility, acceptability, and preliminary effectiveness of the intervention itself. However, it is not yet certain whether this will be its optimal implementation form. For instance, Perfect Fit might be more effective as part of a blended intervention, combined with face-to-face sessions with a healthcare professional. Nonetheless, participants are allowed to use other PA or smoking cessation aids during the study. By assessing the use of these additional aids through questionnaires and post-intervention interviews, we hope to gain preliminary insight into the feasibility and suitability of Perfect Fit as a stand-alone or add-on intervention, as well as the types of interventions it could potentially complement.

## Conclusions

To conclude, the burden on healthcare systems and professionals is substantial, demanding new, cost-effective, accessible and efficient strategies.^12^ mHealth interventions with virtual coaches have the potential to play a complementary role alongside healthcare professionals, enhancing the public health of diverse populations.^10,14^ By combining quantitative and qualitative results on feasibility, acceptability and preliminary effectiveness of Perfect Fit, we hope to gain more insight into whether interventions like Perfect Fit show promise as a multiple health behavior change strategy. The knowledge gained from this study will be used to further develop the intervention, identify improvements and identify how we can promote its usability for different individuals. This knowledge is vital as it will contribute to research on and the development of mHealth interventions with virtual coaches. Further research in this domain could eventually lead to successful interventions to promote public health and thereby reduce the impact of chronic conditions.

## Supplemental Material

sj-docx-1-dhj-10.1177_20552076241300020 - Supplemental material for Protocol of a mixed-methods evaluation of Perfect Fit: A personalized mHealth intervention with a virtual coach to promote smoking cessation and physical activity in adults Supplemental material, sj-docx-1-dhj-10.1177_20552076241300020 for Protocol of a mixed-methods evaluation of Perfect Fit: A personalized mHealth intervention with a virtual coach to promote smoking cessation and physical activity in adults by Milon H. M. van Vliet, Anke Versluis, Niels H. Chavannes, Bouke L. Scheltinga, Nele Albers, Kristell M. Penfornis, Walter Baccinelli, Eline Meijer and in DIGITAL HEALTH

sj-docx-2-dhj-10.1177_20552076241300020 - Supplemental material for Protocol of a mixed-methods evaluation of Perfect Fit: A personalized mHealth intervention with a virtual coach to promote smoking cessation and physical activity in adults Supplemental material, sj-docx-2-dhj-10.1177_20552076241300020 for Protocol of a mixed-methods evaluation of Perfect Fit: A personalized mHealth intervention with a virtual coach to promote smoking cessation and physical activity in adults by Milon H. M. van Vliet, Anke Versluis, Niels H. Chavannes, Bouke L. Scheltinga, Nele Albers, Kristell M. Penfornis, Walter Baccinelli, Eline Meijer and in DIGITAL HEALTH

sj-docx-3-dhj-10.1177_20552076241300020 - Supplemental material for Protocol of a mixed-methods evaluation of Perfect Fit: A personalized mHealth intervention with a virtual coach to promote smoking cessation and physical activity in adults Supplemental material, sj-docx-3-dhj-10.1177_20552076241300020 for Protocol of a mixed-methods evaluation of Perfect Fit: A personalized mHealth intervention with a virtual coach to promote smoking cessation and physical activity in adults by Milon H. M. van Vliet, Anke Versluis, Niels H. Chavannes, Bouke L. Scheltinga, Nele Albers, Kristell M. Penfornis, Walter Baccinelli, Eline Meijer and in DIGITAL HEALTH

sj-docx-4-dhj-10.1177_20552076241300020 - Supplemental material for Protocol of a mixed-methods evaluation of Perfect Fit: A personalized mHealth intervention with a virtual coach to promote smoking cessation and physical activity in adults Supplemental material, sj-docx-4-dhj-10.1177_20552076241300020 for Protocol of a mixed-methods evaluation of Perfect Fit: A personalized mHealth intervention with a virtual coach to promote smoking cessation and physical activity in adults by Milon H. M. van Vliet, Anke Versluis, Niels H. Chavannes, Bouke L. Scheltinga, Nele Albers, Kristell M. Penfornis, Walter Baccinelli, Eline Meijer and in DIGITAL HEALTH

## Notes

a.
https://niceday.app/

b.
https://github.com/PerfectFit-project/virtual-coach-main

c.
https://perfectfit-onderzoek.nl/
