# Supplementary material for: Protocol of a mixed-methods evaluation of Perfect Fit: A personalized mHealth intervention with a virtual coach to promote smoking cessation and physical activity in adults
Source: Digit Health. 2024 Dec 5;10:20552076241300020. doi: 10.1177/20552076241300020 (PMC11618927; doi:10.1177/20552076241300020)
Supplement: sj-docx-1-dhj-10.1177_20552076241300020 - Supplemental material for Protocol of a mixed-methods evaluation of Perfect Fit: A personalized mHealth intervention with a virtual coach to promote smoking cessation and physical activity in adults [file sj-docx-1-dhj-10.1177_20552076241300020.docx]

**Appendix A.** *Description, aims and theoretical basis of Perfect Fit components*

| ***Table A1***  *System-initiated components of Perfect Fit and their intended influences on determinants of physical activity enhancement and smoking cessation.* | | | | |
| --- | --- | --- | --- | --- |
| **Week** | **Component** | **Description** | **Aims and determinants** | **Theory, evidence and/or BCT^a^** |
| ***Preparation phase*** (1.5-3 weeks, based on user preference) | | | | |
| 1 | Introduction video preparation phase | The introduction video of the preparation phase…  … introduces the Perfect Fit program and coach Sam.  … explains that users are interacting with a computer-based virtual coach and not an actual human being. It also explains the importance of contacting a health care professional (e.g., general practitioner) in case of medical problems and the researchers in case of technical problems.  … explains the goals and content of Perfect Fit, and what is expected from the users during Perfect Fit. Also, it explains the beneficial effects of targeting smoking cessation and physical activity (PA) enhancement simultaneously (e.g., quitting smoking increases fitness, which makes PA easier, and PA decreases nicotine cravings).  … explains how to use the NiceDay app (i.e., the smartphone app through which users can interact with the coach) and how to communicate with coach Sam (from a technical point of view).  [Link to 'Introduction video preparation phase'](https://eur03.safelinks.protection.outlook.com/?url=https%3A%2F%2Fvideo.leidenuniv.nl%2Fmedia%2Ft%2F1_hueym3k9&data=05%7C01%7Ca.versluis%40lumc.nl%7C4f9397a255a74e7a602908db563ac87a%7Cc4048c4fdd544cbd80495457aacd2fb8%7C0%7C0%7C638198582694372717%7CUnknown%7CTWFpbGZsb3d8eyJWIjoiMC4wLjAwMDAiLCJQIjoiV2luMzIiLCJBTiI6Ik1haWwiLCJXVCI6Mn0%3D%7C3000%7C%7C%7C&sdata=8doPepPX9FsmB7NmYSRea9Mp%2BxIXl%2BXx7RziL%2B%2BHgFo%3D&reserved=0) (in Dutch) | Introduce participants to the Perfect Fit program, manage expectations by explaining that the coach is computer-based and explaining how to use the app, inform about consequences of health behavior, and educate on benefits of quitting smoking and increasing PA simultaneously. | - ^1-5^ - ^b^*BCT 5.1/5.3/5.6:* Information about consequences of behavior^6-8^ - ^c^*BCT RC4:* Explain expectations regarding treatment program^7^ |
|  | Profile creation dialog | In the profile creation dialog…  … users enter their participant code received from the researchers, to ensure anonymization.  … users are asked which day and day-part of the week they most often have time to chat with coach Sam, to ensure that most Perfect Fit dialogs are triggered on that day.  … users are asked about their preference for running or walking, their current level of PA, and their self-efficacy for running/walking. Also, they are presented with some testimonials and asked to rate these in perceived similarity to themselves. All this data will be used to personalize the testimonials in the goal-setting dialog. | Collect users’ input to tailor content and timing of intervention and interactions with coach Sam to increase personal relevance, feeling of control, and motivation. | - ^9-12^ - *BCT RD1:* Tailor interactions appropriately^7^ - *BCT RD2:* Emphasize choice^7^ |
| Medication video | The medication video…  … explains how nicotine cravings and withdrawal symptoms work.  … explains the importance of abrupt cessation (instead of cutting down gradually) and explains how nicotine replacement therapy or smoking cessation medication could help to quit smoking (in general; no medical advice is given in Perfect Fit).  … briefly explains what nicotine replacement therapy does and how to use it (in general). Users are advised to read the package leaflet when using it and ask any questions they might have to their general practitioner.  … briefly explains what smoking cessation medication does and how long it takes before it is effective (in general). Users are advised to make an appointment with their general practitioner when they want to use medication.  … prompts users to think about whether they want to use nicotine replacement therapy or medication and encourages them to arrange this as soon as possible.  [Link to 'Medication video'](https://eur03.safelinks.protection.outlook.com/?url=https%3A%2F%2Fvideo.leidenuniv.nl%2Fmedia%2Ft%2F1_uu7estu1&data=05%7C01%7Ca.versluis%40lumc.nl%7C4f9397a255a74e7a602908db563ac87a%7Cc4048c4fdd544cbd80495457aacd2fb8%7C0%7C0%7C638198582694372717%7CUnknown%7CTWFpbGZsb3d8eyJWIjoiMC4wLjAwMDAiLCJQIjoiV2luMzIiLCJBTiI6Ik1haWwiLCJXVCI6Mn0%3D%7C3000%7C%7C%7C&sdata=DyY0mhDDlqz9V9C%2B5aVcuCFCUYAjRgfuHmJ%2F07IUqcM%3D&reserved=0) (in Dutch) | Educate on use of nicotine replacement therapy and medication for quitting smoking to facilitate effective use of adjuvant activities and enhance self-regulatory skills by facilitating action planning. | - StopCoach app^13^ - ^5^ - *BCT 1.4:* Action planning^6-8^ - *BCT 11.1:* Pharmacological support^6, 7^ |  |
|  | Tracking current behavior video | The tracking current behavior video…  … explains the rationale behind self-monitoring of current behavior.  … explains that wearing the smartwatch is important so that their PA can be automatically tracked. It also explains that coach Sam will send chat messages to inform users about their step count and that users can also check the smartwatch themselves to see their current step count.  … asks users to track their smoking behavior for at least 3 days using the ‘cigarette tracking’ functionality of the NiceDay app.  … explains how self-monitoring can also provide insight in difficult moments (i.e., HRSs) or situations in which it is easier to resist smoking or be physically active (e.g., certain times of the day, certain locations).  [Link to 'Tracking current behavior video'](https://video.leidenuniv.nl/media/t/1_q3ub2axv) (in Dutch) | Explain rationale behind and prompt self-monitoring to gain insight into current behavior, and increase awareness of HRSs/barriers. | - Relapse Prevention Model^14^ - ^1^ - *BCT 2.3:* Self-monitoring of behavior^6-8^ - *BCT 4.2:* Information about antecedents^6^ |
|  | Future-self video (long) | The future-self video (long)…  … explains the rationale behind the future-self exercise.  … instructs users to start with a practice imagery exercise (i.e., envisioning holding an orange and thinking about the shape, color, structure etc.).  … asks users to envision their current self regarding smoking and PA.  … asks users to envision their undesired future self, when they are still smoking and have not increased their PA. Afterward, users are encouraged to write about this exercise and select (from provided pictures in the information booklet)/search for (themselves, e.g. online, own photos) a picture that matches their undesired future self.  … asks users to envision their desired future self, when they have quit smoking and increased their PA. Afterward, users are encouraged to write about this exercise and select/search for a picture that matches their desired future self.  … asks users to write down differences and similarities between their undesired and desired self and which of the future selves they want to become. They are also asked to write down some first steps they can take towards their future self.  … gives users the option to also envision taking this first small step towards their future self, to evaluate if this step will help them reach their goals.  [Link to 'Future-self video (long)'](https://video.leidenuniv.nl/media/t/1_phg9aofa) (in Dutch) | Strengthen (weaken) the desired (undesired) future identities associated with PA and smoking, prompt reflection on discrepancy between current behavior and future self, enhance self-regulatory skills by facilitating action planning, and increase confidence and motivation to become (avoid) this future self. | - Theories of identity change^15-18^ - ^1, 5, 19^ - Based on results of an ongoing focus group study conducted within the Perfect Fit consortium. - *BCT 1.4:* Action planning^6-8^ - *BCT 1.6:* Discrepancy between current behavior and goal^6, 8^ - *BCT 9.3:* Comparative imagining of future outcomes^6^ - *BCT 13.5:* Identity associated with changed behavior^6, 7^ |
| 2 | Goal-setting dialog | In the goal-setting dialog…  … the rationale behind goal setting is explained.  … the importance of abrupt cessation is explained and users are guided in setting a quit date.  … users are guided in making an action plan to quit smoking and writing down the first steps they can take to quit smoking.  … users are informed about recommended PA levels and injury prevention and read personalized testimonials from other people working on behavioral goals. These testimonials are matched with user characteristics (e.g., initial self-efficacy for running or walking, perceived similarity to examples of people who wrote testimonials), thereby creating personalized vicarious experiences.  …users are guided through setting a SMART (i.e., Specific, Measurable, Achievable, Relevant and Time-Bound) long-term PA goal.  … it is explained that within Perfect Fit the focus is on daily personalized step goals and how this is relevant to their set long-term PA goal.  … users are informed of their first personalized step goal (for the next day) and guided in creating an action plan to achieve daily step goals, including writing down the first steps they can take to increase their daily step count. | Advise on how to perform the behavior, enhance self-regulatory skills by facilitating goal-setting and action planning, and show personalized testimonials to elicit social comparison and realistic expectations (which might help set attainable goals). | - ^5, 9, 11, 20-22^ - *BCT 1.1/1.3:* Goal setting^6-8^ - *BCT 1.4:* Action planning^6-8^ - *BCT 4.1:*  Instruction on how to perform the behavior^6, 8^ - *BCT 6.2:* Social comparison^6, 8^ |
|  | Sensor technology feedback message | After the goal-setting dialog is completed, users receive a message/notification every morning with their personalized daily step goal. This step goal is based on the 7^th^ highest amount of steps during the previous 9 days. Users are also informed about their current step count.  When users have a step count of ≥ 8000 steps on 4 out of the previous 7 days and their total step count from the past 7 days ≥ 56.000 steps, they are encouraged to also increase their weekly minutes of intensive PA (next to reaching their daily step goal). These users will receive daily PA-goal messages about step count and minutes of intensive PA. | Provide feedback on behavior and create personalized small achievable tasks (i.e. short-term goals) to boost self-efficacy and motivation, and prompt desired behavior. | - ^23^ - *BCT 2.2:* Feedback on behavior^6-8^ - *BCT 7.1: P*rompts and cues^6^ - *BCT 8.7:* Graded tasks ^6-8^ - *BCT RD1:* Tailor interactions appropriately^7^ |
|  | First aid kit explanation video | The first aid kit explanation video explains to users how they can add short activities (see ‘Short (optional) activities’ Table A2) to their personal first aid kit and how they can use this first aid kit during HRSs (see ‘First aid kit dialog’ Table A2).  [Link to 'First aid kit explanation video'](https://video.leidenuniv.nl/media/t/1_p2wvnkdf) (in Dutch) | Explain how the first aid kit works. | - Relapse Prevention Model^14^ - *BCT RC4:* Explain expectations regarding treatment program^7^ |
|  | General activity dialog | In the general activity dialog…  … users are asked how useful they found the previously completed short activity (see ‘Short (optional) activities’ Table A2) on a scale of 0 to 10. The five activities with the highest usefulness ratings will be part of their personalized first aid kit (see ‘First aid kit dialog’ Table A2).  … in case the previously completed short activity will be added to the first aid kit, users are informed about this and asked if they want to add any additional notes to the activity (e.g., the most important thing they learned from the activity).  … users are asked which activity they like to do next, which they can choose from a list of short activities.  … the coach sends users a tailored persuasive message (see ‘Tailored persuasive messages’ Table A3) based on a reinforcement algorithm to encourage them to complete the next activity. This is followed by a description of the activity. | Collect users’ input to provide personalized (re)lapse prevention strategies and tailored persuasive messages to increase personal relevance, feeling of control, and motivation. | - Relapse Prevention Model^14^ - ^24-26^ - *BCT RD1:* Tailor interactions appropriately^7^ - *BCT RD2:* Emphasize choice^7^ |
|  | Short activities | In general, the short activities are optional and can be initiated by users themselves whenever they need it. However, some of the short activities are proposed by the coach at certain time points, as these activities are considered important for the behavior change process. This concerns the activities ‘Choose your sports or PA’ as preparation for the goal-setting dialog, ‘Do it differently’ and ‘Changes in your body’ to provide some extra support just after the quit date, and ‘Future-self video (short)’ to encourage users to repeat the future-self exercise multiple times during Perfect Fit. See ‘Short (optional) activities’ Table A2 for more information about the activities. | See aims and determinants ‘Short (optional) activities’ Table A2. | See literature ‘Short (optional) activities’ Table A2. |
| ***Execution phase*** (12 weeks) | | | | |
| 1 | Introduction video execution phase | The introduction video of the preparation phase…  … congratulates users on completing the preparation phase and explains the goals and content of the execution phase. Thereby also discussing what is expected from the users during this phase.  … emphasizes the beneficial effects of targeting smoking cessation and PA enhancement simultaneously.  … normalizes that behavior change can sometimes be difficult and that users can encounter HRSs. It also explains that (re)lapses are learning experiences rather than failures and that every step towards their goals is a success.  … explains how users can initiate certain dialogs, short activities or videos by sending “commands” to the coach.  [Link to 'Introduction video execution phase'](https://video.leidenuniv.nl/media/t/1_pupwgup1) (in Dutch) | Introduce participants to the execution phase of the program, compliment on progress and effort (rather than solely focusing on results), educate on benefits of quitting smoking and increasing PA simultaneously, provide reassurance that negative experiences in a behavior change process are normal and time-limited, and provide instructions for interaction with coach Sam. | - Relapse Prevention Model^14^ - ^1-5^ - *BCT 10.4:* Social reward for effort or progress^6-8^ - *BCT RC4:* Explain expectations regarding treatment program^7^ - *BCT RC10:* Provide reassurance^7^ |
| 1-12 | Sensor technology feedback message | See description ‘Sensor technology feedback’ Table A1, Preparation phase. | See aims and determinants ‘Sensor technology feedback’ Table A1, Preparation phase. | See literature ‘Sensor technology feedback’ Table A1, Preparation phase. |
|  | General activity dialog | See description ‘General activity dialog’ Table A1, Preparation phase. | See aims and determinants ‘General activity dialog’ Table A1, Preparation phase. | See literature ‘General activity dialog’ Table A1, Preparation phase. |
|  | Weekly reflection dialog | In the weekly reflection dialog…  … users are congratulated on completing the corresponding week from the execution phase.  … the coach provides users with an overview of their behavior of the previous week and users are asked to reflect on this (e.g., in terms of quitting smoking and increasing their PA). It is explained why insight into HRSs can be useful.  … users are encouraged to think and tell the coach about HRSs (i.e., difficult moments and actual lapse or relapse) they encountered during the past week. Characteristics of the HRSs (e.g., location, feelings) that users encounter during Perfect Fit are summarized in a graph.  … HRSs and (re)lapses are normalized, users are encouraged to be persistent and celebrate (small) achievements, and the coach provides tips on coping with HRSs and/or (re)lapses.  … users are reminded about their goals and asked to look ahead to the upcoming week to identify potential HRSs they could encounter, thereby using the graph summarizing their personal HRSs.  … users are encouraged to think about strategies to cope with these upcoming HRSs and to create a plan for the upcoming week (including implementation intentions: “When I go to my friend’s party next week, I will ask them to help me not to smoke”). | Compliment on progress and effort, provide feedback on behavior, prompt reviewing behavioral goals, collect users’ input to provide a graph summarizing personal HRSs, increase awareness of HRSs/barriers and teach how to overcome these to prevent (re)lapse, provide reassurance that negative experiences in a behavior change process are normal and time-limited, and enhance self-regulatory skills by facilitating action planning. | - Relapse Prevention Model^14^ - ^1, 5^ - *BCT 1.2:* Barrier identification and problem solving/ relapse prevention^6-8^ - *BCT 1.4:* Action planning^6-8^ - *BCT 1.5:* Review behavior goals^6-8^ - *BCT 2.2:* Feedback on behavior^6-8^ - *BCT 4.2:* Information about antecedents^6^ - *BCT 10.4:* Social reward for effort or progress^6-8^ - *BCT RC10:* Provide reassurance^7^ - *BCT RD1:* Tailor interactions appropriately^7^ |
| ***Anytime during the intervention when users’ daily step count is <8000, and their personalized daily step goal is not met for 3 consecutive days or 4 out of 5 consecutive days.*** | | | | |
|  | HRS and (re)lapse dialog: PA | In the PA HRS and (re)lapse dialog…  … HRSs and lapses are normalized and it is explained why insight in HRSs can be useful.  … users are asked what makes it difficult to be active and encouraged to tell the coach about HRSs they encountered during the past week. Characteristics of the HRSs (e.g., location, feelings) that users encounter during Perfect Fit are summarized in a graph.  … users are encouraged to keep working on their PA goals and the coach provides tips on coping with HRSs and/or lapses.  … the coach gives users the option to 1) look at their first aid kit (see ‘First aid kit dialog’ Table A2), 2) choose an activity from the list of optional short activities (see ‘Short (optional) activities’ Table A2), or 3) end the conversation and continue working on their goals. | Prompt reflection on discrepancy between current behavior and set goals, collect users’ input to provide a graph summarizing personal HRSs, increase awareness of HRSs/barriers and teach how to overcome these (to prevent lapse), and provide reassurance that negative experiences in increasing PA are normal and time-limited. | - Relapse Prevention Model^14^ - ^1, 5^ - *BCT 1.2:* Barrier identification and problem solving/ relapse prevention^6-8^ - *BCT 1.6:* Discrepancy between current behavior and goal^6, 8^ - *BCT 4.2:* Information about antecedents^6^ - *BCT RC10:* Provide reassurance^7^ - *BCT RD1:* Tailor interactions appropriately^7^ |
| ***Closing phase*** (1 dialog) | | | | |
|  | Closing dialog | In the closing dialog…  … users are congratulated on completing Perfect Fit and encouraged to reward themselves for this achievement. It is suggested that they could use the money that they saved by quitting smoking to reward themselves.  … the coach reminds users about the set goals at the start of Perfect Fit and shows users their progress.  … the coach compliments users who quit smoking and positively reinforces users who are still in the process of quitting smoking (i.e., lapsed or relapsed in the previous month) to keep going.  … users are asked to reflect on whether they (partly) achieved their goals and how close they currently are to their desired future self (see ‘Future-self video (long)’ Table A1, Preparation phase).  … the coach shows users the graph summarizing their personal HRSs (see ‘Weekly reflection dialog’ Table A1, Execution phase), their first aid kit with the five activities they found most useful (see ‘First aid kit dialog’ Table A2) and explains that they can find all the short (optional) activities on the Perfect Fit website after the intervention (<https://perfectfit-onderzoek.nl/informatie>).  … users are guided through making a relapse prevention plan and the coach provides some final tips.  … the coach asks users to rate Perfect Fit and provide feedback on how to make Perfect Fit better. | Compliment on progress, effort and results, encourage self-rewarding and celebrating achievements to build confidence and boost motivation, make positive consequences of health behavior change more salient by prompting users to focus on money they saved by quitting smoking, provide feedback on behavior, and prompt reviewing behavioral goals, collect users’ input to provide a graph summarizing personal HRSs, and prompt making a relapse prevention plan on basis of personal HRSs/barriers and learned coping strategies. | - Relapse Prevention Model^14^ - *BCT 1.2:* Barrier identification and problem solving/ relapse prevention^6-8^ - *BCT 1.5:* Review behavior goals^6-8^ - *BCT 2.2:* Feedback on behavior^6-8^ - *BCT 4.2:* Information about antecedents^6^ - *BCT 5.2:* Salience of consequences^6, 7^ - *BCT 10.4:* Social reward for effort or progress and behavior^6-8^ - *BCT 10.7/10.9:* self-incentive and/or self-reward^6-8^ - *BCT RD1:* Tailor interactions appropriately^7^ |
| The source code of the Perfect Fit system^[[1]](#footnote-1)^ is publicly available.^27^  The dialogs and short (optional) activities are publicly available (in Dutch) on OSF.^[[2]](#footnote-2)^  ^a^Intervention components were also chosen and created in collaboration with end-users and experts, see Versluis et al.^28^ for a more detailed description.  ^b^BCTs numbered from 1.1 to 13.5 refer to the general behavior change technique taxonomy (v1) of 93 techniques of Michie et al.^6^  ^c^BCTs with codes RC and RD refer to the taxonomy of behavior change techniques used for smoking cessation support of Michie et al.^7^  BCT = behavior change technique; PA = Physical activity; HRS = high risk situation | | | | |

| ***Table A2***  *User-initiated components of Perfect Fit and their intended influences on determinants of physical activity enhancement and smoking cessation.* | | | |
| --- | --- | --- | --- |
| **Component** | **Description** | **Aims and determinants** | **Theory, evidence and/or BCT^a^** |
| Medication video | Users can indicate any time during the intervention if they want to re-watch the medication video. See description ‘Medication video’ Table A1, Preparation phase. | See aims and determinants ‘Medication video’ Table A1, Preparation phase. | See literature ‘Medication video’ Table A1, Preparation phase. |
| Sensor technology feedback message | Users can send the word ‘goal’ to the coach to receive a message with their personalized PA goals. See description ‘Sensor technology feedback’ Table A1, Preparation phase. | See aims and determinants ‘Sensor technology feedback’ Table A1, Preparation phase. | See literature ‘Sensor technology feedback’ Table A1, Preparation phase. |
| First aid kit explanation video | Users can indicate any time during the intervention if they want to re-watch the first aid kit explanation video. See description ‘First aid kit explanation video’ Table A1, Preparation phase. | See aims and determinants ‘First aid kit explanation video’ Table A1, Preparation phase. | See literature ‘First aid kit explanation video’ Table A1, Preparation phase. |
| General activity dialog | Users can send the word ‘Activity’ to the coach to start the General activity dialog. See description ‘General activity dialog’ Table A1, Preparation phase. | See aims and determinants ‘General activity dialog’ Table A1, Preparation phase. | See literature ‘General activity dialog’ Table A1, Preparation phase. |
| First aid kit dialog | Users can send the words ‘first aid kit’ to the coach to see their personalized first aid kit with the five activities (see ‘Short (optional) activities’ Table A2) that they perceive as most useful, including any additional notes they made (see ‘General activity dialog’ Table A1, Preparation phase). Users are recommended to consult this first aid kit during HRSs. | Increase awareness of HRSs/barriers, and collect users’ input to provide personalized (re)lapse prevention strategies to increase personal relevance, feeling of control, and motivation. | - Relapse Prevention Model^14^ - ^1, 5^ - ^b^*BCT 1.2:* Barrier identification and problem solving/ relapse prevention^6-8^ - ^c^*BCT RD1:* Tailor interactions appropriately^7^ - *BCT RD2:* Emphasize choice^7^ |
| HRS and (re)lapse dialog: PA and smoking | Users can send the word ‘help’ to the coach to start the HRS and (re)lapse dialog. At the beginning of the dialog users can indicate if they want help with PA enhancement or smoking cessation and if it concerns a difficult moment (i.e., HRS), lapse or relapse. The dialog is tailored accordingly. In case of smoking relapse, users will receive support from the coach, can rewatch the medication video and/or (re)do short activities, and can set a new quit date. For further description see ‘HRS and (re)lapse dialog: PA’ Table A1. | See aims and determinants ‘HRS and (re)lapse dialog: PA’ Table A1. | See literature ‘HRS and (re)lapse dialog: PA’ Table A1. |
| ***Short (optional) activities*** | | | |
| *Self-related activities^[[3]](#footnote-3)^* | | | |
| Future-self video (short) | Short version of the ‘Future-self video (long)’ (see Table A1, Preparation phase) to encourage users to repeat the future-self exercise. In this video, users are asked to envision their undesired future self and to envision, write about, and search for pictures that match their desired future self. They are also asked to write down and envision some first small steps they can take towards their future self.  [Link to 'Future-self video (short)'](https://eur03.safelinks.protection.outlook.com/?url=https%3A%2F%2Fvideo.leidenuniv.nl%2Fmedia%2Ft%2F1_7auemsl7&data=05%7C01%7Ca.versluis%40lumc.nl%7C4f9397a255a74e7a602908db563ac87a%7Cc4048c4fdd544cbd80495457aacd2fb8%7C0%7C0%7C638198582694372717%7CUnknown%7CTWFpbGZsb3d8eyJWIjoiMC4wLjAwMDAiLCJQIjoiV2luMzIiLCJBTiI6Ik1haWwiLCJXVCI6Mn0%3D%7C3000%7C%7C%7C&sdata=iMrqzRDYgEt8jAEZfUm2QB4AlYGkKWSnAgjCiLoQ8Fs%3D&reserved=0) | See aims and determinants ‘Future-self video (long)’ Table A1, Preparation phase. | See literature ‘Future-self video (long)’ Table A1, Preparation phase. |
| Feedback from friends and family on the new me | Prompt users to envision how friends/family will react when they tell them that they want to become their desired future self and let users think about how they could cope with negative reactions of friends/family. This will help lower the fear of judgment from friends/family and build confidence in telling others about who they (do not) want to become. | Provide/elicit social support, and increase self-efficacy and motivation to become desired future self. | - Theories of identity change^15-18^ - ^29^ - *BCT 3:* Social support^6-8^ - *BCT 13.5:* Identity associated with changed behavior^6, 7^ |
| Taking photos of yourself | Prompt users to take a selfie/photo of themselves at several stages of the intervention to see differences in their appearance over time to show the positive changes that can result from quitting smoking and increasing PA (e.g., better looking skin). | Inform about positive consequences of health behavior change and make these more salient by photographing them to boost motivation. | - StopCoach app^13^ - *BCT 5.1/5.3/5.6:* Information about consequences of behavior^6-8^ - *BCT 5.2:* Salience of consequences^6, 7^ |
| You as a role model | Ask users to think about themselves as a role model to others and about their successes in changing their behaviors since the start of the intervention. Let them think about what helped them changing their behaviors and what did not, and how they handled difficult moments. Subsequently, prompt users to think about what they would advise others in their situation. | Prompt identification as a role model and let users think about past successes and effective coping strategies to boost self-efficacy and motivation. | - *BCT 13.1:* Identification as a role model^6, 8^ - *BCT 15.3:* Focus on past success^6, 8^ |
| Persistence | Explain to users that it can be difficult to tell others that they want to quit smoking and/or increase their PA, especially if they have a lot of smokers/physically inactive people in their social network. Provide tips on how to handle situations in which this can be difficult and on searching for social support from other people (who want to quit smoking and/or increase their PA). | Provide/elicit social support and increase self-efficacy. | - StopCoach app^13^ - ^1, 5^ - *BCT 3:* Social support^6-8^ |
| *Educational activities* | | | |
| Changes in your body | Educate users about physical transformations linked to quitting smoking (e.g., breathing becomes easier, but also withdrawal symptoms like difficulty sleeping) and being more physically active (e.g., improved mood, but also muscle soreness). Also, inform users about the synergy between smoking cessation and PA increase. | Inform about consequences of health behavior, prepare for physical transformations, and provide reassurance that negative experiences associated with increasing PA or quitting smoking are normal and time-limited. | - StopCoach app^13^ - ^1-5, 30^ - *BCT 5.1/5.3/5.6:* Information about consequences of behavior^6-8^ - *BCT RC10:* Provide reassurance^7^ |
| Tips for PA | Educate users on the importance of decreasing sedentary behavior and increasing PA, and recommended levels of PA. | Inform about consequences of health behavior, prompt reflection on current level of PA and advise on how to perform the behavior. | - ^1, 30^ - *BCT 4.1:* Instruction on how to perform the behavior^6, 8^ - *BCT 5.1/5.3/5.6:* Information about consequences of behavior^6-8^ |
| Healthy eating | Explain to users why people can gain weight while quitting smoking. Educate users about a healthy diet and strategies to prevent gaining weight while quitting smoking (e.g., being physically active). | Inform about consequences of health behavior, provide reassurance that negative experiences associated with quitting smoking are normal and time-limited, and focus on specific potential barrier (i.e., gaining weight) and teach how to overcome this to prevent (re)lapse. | - StopCoach app^13^ - *BCT 1.2:* Barrier identification and problem solving/ relapse prevention^6-8^ - *BCT 4.1:* Instruction on how to perform the behavior^6, 8^ - *BCT 5.1/5.3/5.6:* Information about consequences of behavior^6-8^ - *BCT RC10:* Provide reassurance^7^ |
| Improving sleep hygiene | Explain to users why people can experience sleeping problems as a consequence of quitting smoking and give advice for improving sleep hygiene. | Inform about consequences of health behavior, provide reassurance that negative experiences associated with increasing PA or quitting smoking are normal, and focus on specific potential barrier (i.e., sleeping problems) and teach how to overcome this to prevent (re)lapse. | - StopCoach app^13^ - ^31-33^ - *BCT 1.2:* Barrier identification and problem solving/ relapse prevention^6-8^ - *BCT 4.1:* Instruction on how to perform the behavior^6, 8^ - *BCT 5.1/5.3/5.6:* Information about consequences of behavior^6-8^ - *BCT RC10:* Provide reassurance^7^ |
| Coffee and alcohol consumption | Educate users on the effect of smoking on caffeine and the impact of caffeine and alcohol on smoking cessation and PA performance and let them think about/provide strategies for dealing with this. | Inform about consequences of health behavior and focus on specific potential barriers (i.e., consuming a high amount of caffeine and alcohol) and teach how to overcome these to prevent (re)lapse. | - StopCoach app^13^ - ^34-36^ - *BCT 1.2:* Barrier identification and problem solving/ relapse prevention^6-8^ - *BCT 4.1:* Instruction on how to perform the behavior^6, 8^ - *BCT 5.1/5.3/5.6:* Information about consequences of behavior^6-8^ |
| Managing stress | Educate users on the impact of stress on smoking cessation and PA performance and let them think about/provide strategies to cope with stress. | Inform about consequences of health behavior and focus on specific potential barrier (i.e., experiencing stress) and teach how to overcome this to prevent (re)lapse. | - ^37^ - *BCT 1.2:* Barrier identification and problem solving/ relapse prevention^6-8^ - *BCT 4.1:* Instruction on how to perform the behavior^6, 8^ - *BCT 5.1/5.3/5.6:* Information about consequences of behavior^6-8^ |
| Your body becomes healthier | Inform users about negative symptoms (e.g., nicotine withdrawal symptoms, muscle soreness) they could experience as a result of quitting smoking and increasing PA. Subsequently, turn this into something positive by explaining that these are signs that the body is becoming healthier. Also, provide tips on how to deal with these symptoms. | Inform about consequences of health behavior, provide reassurance that negative symptoms associated with increasing PA or quitting smoking are normal and time-limited, generate positive beliefs about negative symptoms, and focus on specific potential barriers (i.e., negative symptoms) and teach how to overcome these to prevent (re)lapse. | - StopCoach app^13^ - ^1, 5^ - *BCT 1.2:* Barrier identification and problem solving/ relapse prevention^6-8^ - *BCT 4.1:* Instruction on how to perform the behavior^6, 8^ - *BCT 5.1/5.3/5.6:* Information about consequences of behavior^6-8^ - *BCT RC10:* Provide reassurance^7^ |
| *Motivational and self-efficacy activities* | | | |
| Your agreements | Ask users to commit to reaching their quit-smoking and PA enhancement goals. This will be encouraged by creating a personal rule not to smoke and/or to increase PA as part of a behavioral contract. Users will be asked to generate motivational slogans. | Construct personal rule/behavioral contract to generate commitment to change behavior and boost motivation. | - ^1, 5, 18^ - *BCT 1.8/1.9:* Behavioral contract/ commitment^6-8^ |
| Benefits of quitting smoking and increasing PA | Ask users to write down reasons to quit smoking and increase PA, and formulate positive expectations of improving these behaviors. | Identify benefits of the behavior change to boost motivation. | - StopCoach app^13^ - ^1, 5, 38^ - *BCT 9.2:* Pros and cons/reasons to change behavior^6-8^ |
| Barriers | Let users think about barriers to quit smoking and increase PA (e.g., ‘I don’t have running shoes’) and think about how barriers can be tackled (e.g., ‘Buy running shoes’). | Increase awareness of HRSs/barriers and teach how to overcome these to prevent (re)lapse and to increase self-efficacy. | - Relapse Prevention Model^14^ - ^1, 5, 39, 40^ - *BCT 1.2:* Barrier identification and problem solving/ relapse prevention^6-8^ |
| Fighting match | Let users visualize quitting smoking and/or increasing PA as a battle (e.g., boxing or swimming competition) and imagine winning this battle. | Encourage users to visualize winning “the battle against their undesired behaviors” to boost self-efficacy and motivation. | - ^1, 5^ - *BCT 15.1:* Mental rehearsal of successful performance^6^ |
| Positive phrases | Explain the positive effect of positive thoughts on behavior change. Prompt users to use encouraging, positive self-talk about achieving their behavioral goals (e.g., “I am capable of quitting smoking.”, “I can achieve my weekly goal of walking 15 minutes outside every day.”). | Prompt positive self-talk to boost self-efficacy and motivation. | - ^41, 42^ - *BCT 15.4:* Self-talk^6, 8^ |
| Reflecting on your achievements | Have users think about successes which are unrelated to smoking or PA (e.g., learning a language, decorating your living room) and on successes related to quitting smoking or increasing PA. Let users think about what worked and what did not in achieving these successes. | Let users think about past successes to boost self-efficacy and motivation. | - *BCT 15.3:* Focus on past success^6, 8^ |
| *Practical activities* | | | |
| Choose your sports or PA | Encourage users to consider what type of PA they would like to do and empower them to create a plan accordingly. Emphasize the importance of selecting PA that they enjoy and suggest exploring nearby gyms/pools/clubs, looking up workout videos and/or creating detailed routes for running or walking. | Advise on how to perform PA, encourage selecting PA that they enjoy to minimize demands on mental resources, and enhance self-regulatory skills by facilitating action planning. | - ^1^ - *BCT 1.4:* Action planning^6-8^ - *BCT 4.1:* Instruction on how to perform the behavior^6, 8^ - *BCT 11.2/11.3:* Reducing negative emotions/ Conserving mental resources^6-8^ |
| Restructuring your environment | Prompt users to alter the environment in ways that help them reaching their behavioral goals. For example, they could take their running shoes to work (PA), or throw away all their cigarettes and wash their curtains (smoking cessation). | Advise on avoiding cues and/or changing the environment to trigger the desired behavior and avoid triggering the undesired behavior, to prevent (re)lapse and enhance self-regulatory skills. | - Relapse Prevention Model^14^ - ^5, 43^ - *BCT 12.1/12.2/ 12.3:* Environmental restructuring/ reducing exposure to cues^6-8^ |
| Relaxation | Let users think about and provide tips for strategies to reduce stress and/or cope with nicotine cravings. For instance, by doing a progressive muscle relaxation or breathing exercise, or other relaxing activities (like gardening, taking a bath, doing something creative). | Support users in coping with stress and nicotine cravings (i.e., HRSs) and teach relaxation techniques to minimize demands on mental resources. | - ^1, 5, 37^ - *BCT 1.2:* Barrier identification and problem solving/ relapse prevention^6-8^ - *BCT 11.2/11.3:* Reducing negative emotions/ Conserving mental resources^6-8^ |
| Do it differently | Prompt users to think about which alternative behaviors they could do instead of smoking (e.g., making a cup of tea, taking a short walk) or instead of low-PA behaviors (e.g., taking the stairs instead of the elevator), and how to implement these alternative behaviors. | Prompt repetition of an alternative behavior to replace the unwanted behavior and enhance self-regulatory skills by facilitating action planning. | - StopCoach app^13^ - ^1, 5^ - *BCT 1.4:* Action planning^6-8^ - *BCT 8.2/8.4:* Behavior substitution/habit reversal^6^ |
| *Reward activities* | | | |
| Celebrate your success | Encourage users to celebrate or self-reward (small) milestones and progress during Perfect Fit and let them think about how they will reward themselves (e.g., buying a new book or celebrating success with a friend). | Encourage self-reward and celebrating (small) achievements to boost self-efficacy and motivation. | - StopCoach app^13^ - ^5^ - *BCT 10.7/10.9:* self-incentive and/or self-reward^6-8^ |
| Positive diary | Motivate users to write down, at the end of the day, two or three positive things which happened during the day (e.g., the sun was shining, I walked 20 minutes in the park). | Encourage focusing on positive things and/or achievements to reduce negative emotions and thereby minimize demands on mental resources. | - ^44^ - *BCT 11.2/11.3:* Reducing negative emotions/ Conserving mental resources^6-8^ |
| Reward for completing Perfect Fit | Prompt users to think about how they will reward themselves once they have completed Perfect Fit (e.g., buying a new bike). It is suggested that they could use the money that they saved by quitting smoking. | Encourage users to keep track of money they saved by quitting smoking to make positive consequences of health behavior change more salient, and encourage self-reward and celebrating achievements to boost self-efficacy and motivation. | - StopCoach app^13^ - *BCT 10.7/10.9:* self-incentive and/or self-reward^6-8^ - *BCT 5.2:* Salience of consequences^6, 7^ |
| The source code of the Perfect Fit system is publicly available.^27^  ^a^Intervention components were also chosen and created in collaboration with end-users and experts, see Versluis et al.^28^ for a more detailed description.  ^b^BCTs numbered from 1.2 to to 15.4 refer to the general behavior change technique taxonomy (v1) of 93 techniques of Michie et al.^6^  ^c^BCTs with codes RC and RD refer to the taxonomy of behavior change techniques used for smoking cessation support of Michie et al.^7^  BCT = behavior change technique; PA = Physical activity; HRS = high risk situation | | | |

| ***Table A3***  *Virtual coach and intervention features and intended influences on determinants of the feasibility and acceptability of the intervention.* | | | |
| --- | --- | --- | --- |
| **Features** | **Description** | **Aims and determinants** | **Theory, evidence and/or BCT^a^** |
| System- and user-initiated interaction | The coaching system allows both system- and user-initiated chat conversations. The system-initiated components are designed to align with the behavior change process, ensuring timely delivery and encouraging users to complete key intervention components (see Table A1 for all system-initiated components). User-initiated components allow users to tailor the content and timing of components according to their preferences. For example, users can indicate that they want to do a short activity at a time of their choosing and select their preferred activity from a list. Users can also use commands to re-watch specific videos (e.g., medication video), seek help in coping with HRSs or (re)lapses, consult their personalized first aid kit, or check their current step count and daily step goal (see Table A2 for all user-initiated components). | Make intervention interactive and tailor intervention content and timing to increase personal relevance, feeling of control, and user engagement, while ensuring controllability, safety, and consistency in intervention content delivery. | - ^1^ - ^b^*BCT RD1:* Tailor interactions appropriately^7^ - *BCT RD2:* Emphasize choice^7^ |
| Constrained and unconstrained user input | The coaching system allows users to input pre-programmed responses (e.g., responding with a number from 1 to 5 to indicate how well it went with quitting smoking that week) and free-text responses (e.g., sharing a reflection on successes or setbacks during the week). The coach can also store user input to build on or refer to this information later in the conversation or other conversations. | Tailor intervention content and interactions with coach Sam to increase personal relevance, natural flow of conversations, and user engagement, while ensuring ease of use, controllability, safety, and consistency in intervention content delivery. | - ^1, 45^ - *BCT RC2:* Elicit and answer questions^7^ - *BCT RD1:* Tailor interactions appropriately^7^ |
| Face of animated robot with gender-neutral name | The virtual coach is named ‘Sam’, a gender-neutral name that aims to enhance its human-like qualities while allowing users the freedom to interpret the coach’s gender and/or identity based on their preference. Additionally, the coach is visually represented by the face of an animated robot, ensuring clarity for users that they are interacting with a computer-based virtual coach rather than a human. | Increase personal relevance and build general rapport with coach, while ensuring transparency and realistic expectations. | - ^1, 45^ - Based on results of an ongoing focus group study conducted within the Perfect Fit consortium. |
| Make system as straightforward as possible | The coaching system is simplified as much as possible. For instance, by ensuring a one-time installation, login and connection to the coach, and by sending users notifications that directly lead them to the chat conversation with the coach after clicking on the notification. Also, pre-programmed responses are numbered, enabling users to respond with the corresponding number rather than typing extensive text. | Promote ease of use and user engagement. | - ^5^ - Based on results of an ongoing focus group study conducted within the Perfect Fit consortium. |
| Assist with app installation and wearable | The researchers provide users with an installation booklet detailing how to install the required apps and use the smartwatch. Throughout the intervention, the coach reminds users to wear the smartwatch, offers guidance on reconnecting with the smartwatch when the connection is not working, and encourages users to reach out to the researchers if they encounter persistent problems. | Guide users through the onboarding procedure and explain how to use the apps and wearable to promote ease of use and user engagement. |  |
| Explain how to use system | The coach explains to users how the system works. For instance, videos are used to explain how to use the system during the intervention (e.g., see ‘Introduction video preparation phase’ Table A1, Preparation phase) or certain components (e.g., see ‘First aid kit explanation video’ Table A1, Preparation phase). Also, when presenting a video, the coach guides users on accessing the external link to the video and returning to the chat conversation afterward. Finally, during “free mode”, the coach informs users about the commands the user can use to trigger certain user-initiated dialogs, videos, or activities. | Explain how to use the system to promote ease of use and user engagement. | - ^5^ - *BCT RC4:* Explain expectations regarding treatment program^7^ |
| Be transparent about capability of virtual coach | The virtual coach is transparent about its computer-based nature and its capabilities, to manage expectations. For instance, when users provide free-text responses that the coach does not understand, the coach indicates this and suggests recognized responses to guide users. | Promote transparency and set realistic expectations to increase user engagement. | - ^1^ - *BCT RC4:* Explain expectations regarding treatment program^7^ |
| Use alternative formulations | For dialogs and activities that may occur repeatedly throughout the intervention, the coach employs randomized alternative message formulations for messages to prevent repetition and make chat conversations more natural and human-like. | Prevent boredom by avoiding replication and thereby increase user engagement. | - ^1, 45, 46^ |
| Use reminders | The coach notifies users about scheduled dialogs, videos or activities, as well as encourages continued interaction with the virtual coach. For instance, users receive reminders during the preparation phase to monitor their smoking behavior and wear their smartwatch (see ‘Tracking current behavior video’ Table A1, Preparation phase). Additionally, users are reminded to complete dialogs if inactive for a certain period while in the middle of a dialog. This is important as users easily forget new behaviors when they have not yet become habits. | Use reminders to prompt desired behavior, increase accountability, motivation and user engagement. | - ^1, 5^ - ^c^*BCT 7.1: P*rompts and cues^6^ |
| Explain rationale behind activities | The coach clearly explains the rationale behind intervention components and the benefits they offer, aiming to enhance the perceived usefulness of the content and reduce potential resistance. The coach also explains why certain activities aid users in achieving their behavioral goals and emphasizes the benefits of simultaneously focusing on smoking cessation and PA enhancement. | Explain rationale and importance of activities, to set realistic expectations, and increase perceived usefulness, motivation, and user engagement. | - ^1, 5, 45^ - *BCT RC4:* Explain expectations regarding treatment program^7^ |
| Tailor timing and indicate expected time investment | The timing of intervention components is tailored using various methods: 1) users can initiate some components themselves (see ‘System- and user-initiated interaction’ above), 2) users are asked at the start of the intervention about their preferred time for completing weekly dialogs (see ‘Profile creation dialog’ Table A1, Preparation phase), and 3) short scheduling dialogs are utilized at the start of each new dialog, video or activity. Within these dialogs, the coach indicates the expected time investment for the next dialog/video/activity and proposes alternative timings if the user is unable to invest the foreseen time, aiming to increase the likelihood of users completing the component in one session. Finally, after the preparation phase, system-initiated dialogs are limited to once per week to prevent users from feeling overwhelmed. | Tailor intervention timing and interactions with coach Sam to increase personal relevance, feeling of control, motivation, and accountability, and adapt to availability user to minimize demands on user and increase user engagement. | - ^1, 5^ - *BCT RD1:* Tailor interactions appropriately^7^ - *BCT RD2:* Emphasize choice^7^ |
| Tailor content | The intervention content is tailored using various methods. For instance, many of the short activities are optional such that users can choose themselves what, and how much, they want to do (see ‘Short (optional) activities’ Table A2). The coach also occasionally inquires if users want to read more about certain topics or receive a more elaborate explanation. Finally, particularly within the short activities, the coach offers external links to additional information, which users can choose whether or not to access. | Tailor intervention content and interactions with coach Sam to increase personal relevance, feeling of control, motivation, and user engagement. | - ^1^ - Based on results of an ongoing focus group study conducted within the Perfect Fit consortium. - *BCT RD1:* Tailor interactions appropriately^7^ - *BCT RD2:* Emphasize choice^7^ |
| Use tailored persuasive messages | The coach uses tailored persuasive messages to motivate users to engage in short activities (see ‘Short (optional) activities’ Table A2). A trained reinforcement learning algorithm is used to select one of five persuasive strategies (i.e., commitment, consensus, authority, action planning, or no persuasion), considering users’ current and future states. The state is based on users’ responses to three questions: 1) whether they feel like wanting to do an activity, 2) whether they have things that prompt or remind them to do an activity, and 3) whether they feel like they need to do an activity. | Collect users’ input to tailor the intervention and interactions with coach Sam to increase personal relevance, motivation, accountability and user engagement. | - ^24, 25^ - *BCT RD1:* Tailor interactions appropriately^7^ |
| Show progress and provide personalized feedback | The coach uses smartwatch data and user input to monitor users’ progress and present it back to them. Moreover, personalized feedback is provided based on this progress. For instance, the coach collects user input on personal HRSs, generates a personalized graph summarizing these situations, and prompts users to reflect on this (see ‘Weekly reflection dialog’ Table A1, Execution phase). Also, personalized feedback is given on users’ PA progress (based on smartwatch data), focusing on increasing daily step count, or for active users (>8000 steps per day), increasing weekly minutes of intensive PA. | Collect users’ input to tailor the intervention and interactions with coach Sam to increase personal relevance, motivation and user engagement and aid setting realistic goals. | - ^1, 5^ - *BCT RD1:* Tailor interactions appropriately^7^ |
| Make use of inclusive communication | The coach aims to use non-suggestive communication that invites diverse interpretations and is adaptable to various contexts and users. For example, this includes providing multiple examples when illustrating statements and avoiding assumptions by using phrasing that is not suggestive such as “Perhaps you notice…”. | Use inclusive communication to increase personal relevance, motivation and user engagement and to build general rapport with coach. | - *BCT RC1:* Build general rapport^7^ - *BCT RD2:* Emphasize choice^7^ |
| Use easy-to-understand language, images, graphs and animated videos | The coach uses B1-level Dutch, emojis, images, and animated videos to enhance accessibility for individuals with lower literacy levels. | Promote ease of use and user engagement. | - ^5^ |
| Use small talk and emojis | The coach uses relational strategies, including small talk and the use of emojis, to make conversations more natural and human-like. For instance, this involves asking users how they have been and expressing pleasure in interacting with them. | Increase mood, user engagement and build general rapport with coach. | - ^5, 45^ - *BCT RC1:* Build general rapport^7^ |
| Show empathy and maintain a supportive attitude | The coach uses relational strategies to show empathy and maintain an encouraging, friendly, and supportive attitude to motivate users. For example, this includes complimenting users on their progress and effort (rather than solely focusing on results), expressing belief in users’ capabilities, emphasizing that setbacks are learning experiences rather than failures, and offering words of encouragement during important moments, such as their first day of quitting smoking. | Use positive message tone to provide social support, increase mood, self-efficacy, motivation, and user engagement, and build general rapport with coach. | - Motivational interviewing^47, 48^ - ^5, 45^ - *BCT 3:* Social support^6-8^ - *BCT 15.1:* Verbal persuasion about capability^6^ - *BCT RC1:* Build general rapport^7^ |
| Promote safety and security | If users provide answers that are not recognized by the coach, the coach indicates this and offers recognized responses to assist users. Also, when users contact the coach during “free mode”, the coach informs users about commands to trigger certain user-initiated intervention components and advises users to consult a professional (e.g., general practitioner or quitting smoking helpline) or a trusted friend if they wish to discuss other matters. In case of technical issues, the coach refers users to the researchers. Users are informed about measures to protect their privacy (e.g. adherence to privacy legislation and implementation of measures to protect their data). | Explain safety and privacy measures to promote transparency and increase trust in safely sharing information, while ensuring controllability and preventing safety risks. | - ^45^ - *BCT RC4:* Explain expectations regarding treatment program^7^ |
| The source code of the Perfect Fit system is publicly available.^27^  ^a^Intervention components were also chosen and created in collaboration with end-users and experts, see Versluis et al.^28^ for a more detailed description.  ^b^BCTs with codes RC and RD refer to the taxonomy of behavior change techniques used for smoking cessation support of Michie et al.^7^  ^c^BCTs numbered from 3 to 15.1 refer to the general behavior change technique taxonomy (v1) of 93 techniques of Michie et al.^6^  BCT = behavior change technique; PA = Physical activity; HRS = high risk situation | | | |

**References**

1. Albers N, Neerincx MA, Penfornis KM, et al. Users’ needs for a digital smoking cessation application and how to address them: A mixed-methods study. *PeerJ* 2022; 10: e13824. DOI: 10.7717/peerj.13824.

2. Berkovitch A, Kivity S, Klempfner R, et al. Time-dependent relation between smoking cessation and improved exercise tolerance in apparently healthy middle-age men and women. *Eur J Prev Cardiol* 2015; 22: 807-814. 2014/05/13. DOI: 10.1177/2047487314535116.

3. Kunicki ZJ, Hallgren M, Uebelacker LA, et al. Examining the effect of exercise on the relationship between affect and cravings among smokers engaged in cessation treatment. *Addict Behav* 2022; 125. DOI: 10.1016/j.addbeh.2021.107156.

4. Priebe CS, Atkinson J and Faulkner G. Run to Quit: An evaluation of a scalable physical activity-based smoking cessation intervention. *Ment Health Phys Act* 2017; 13: 15-21. DOI: 10.1016/j.mhpa.2017.08.001.

5. Michie S, Brown J, Geraghty AWA, et al. Development of StopAdvisor A theory-based interactive internet-based smoking cessation intervention. *Transl Behav Med* 2012; 2: 263-275. DOI: 10.1007/s13142-012-0135-6.

6. Michie S, Richardson M, Johnston M, et al. The Behavior Change Technique Taxonomy (v1) of 93 Hierarchically Clustered Techniques: Building an International Consensus for the Reporting of Behavior Change Interventions. *Ann Behav Med* 2013; 46: 81-95. DOI: 10.1007/s12160-013-9486-6.

7. Michie S, Hyder N, Walia A, et al. Development of a taxonomy of behaviour change techniques used in individual behavioural support for smoking cessation. *Addict Behav* 2011; 36: 315-319. DOI: 10.1016/j.addbeh.2010.11.016.

8. Michie S, Ashford S, Sniehotta FF, et al. A refined taxonomy of behaviour change techniques to help people change their physical activity and healthy eating behaviours: The CALO-RE taxonomy. *Psychol Health* 2011; 26: 1479-1498. DOI: 10.1080/08870446.2010.540664.

9. Albers N, Hizli B, Scheltinga BL, et al. Setting physical activity goals with a virtual coach: vicarious experiences, personalization and acceptance. *Journal of Medical Systems* 2023; 47: 15. DOI: 10.1007/s10916-022-01899-9.

10. Godin G. The Godin-Shephard Leisure-Time Physical Activity Questionnaire. *The Health & Fitness Journal of Canada* 2011; 4: 18-22. DOI: 10.14288/hfjc.v4i1.82.

11. Hizli B, Albers N and Brinkman W-P. Data and code underlying the master thesis: Goal-setting dialogue for physical activity with a virtual coach (Version 1) <https://doi.org/10.4121/20047328.v1> (2022).

12. Mcauley E. Self-Efficacy and the Maintenance of Exercise Participation in Older Adults. *J Behav Med* 1993; 16: 103-113. DOI: 10.1007/Bf00844757.

13. Meijer E, Korst JS, Oosting KG, et al. "At least someone thinks I'm doing well": a real-world evaluation of the quit-smoking app StopCoach for lower socio-economic status smokers. *Addict Sci Clin Prac* 2021; 16: 1-14. DOI: 10.1186/s13722-021-00255-5.

14. Marlatt GA and Donovan DM. *Relapse prevention: Maintenance strategies in the treatment of addictive behaviors*. Guilford press, 2007.

15. Oyserman D and Destin M. Identity-Based Motivation: Implications for Intervention. *Couns Psychol* 2010; 38: 1001-1043. DOI: 10.1177/0011000010374775.

16. Oyserman D and James L. Possible Identities. In: Schwartz SJ, Luyckx K and Vignoles VL (eds) *Handbook of Identity Theory and Research*. New York, NY: Springer New York, 2011, pp.117-145.

17. Markus H and Nurius P. Possible Selves. *Am Psychol* 1986; 41: 954-969. DOI: 10.1037/0003-066x.41.9.954.

18. West R and Brown J. *Theory of Addiction*. 2nd ed. Hoboken, New Jersey: Wiley-Blackwell, 2013.

19. Penfornis KM, Gebhardt WA, Rippe RCA, et al. My future-self has (not) quit smoking: An experimental study into the effect of a future-self intervention on smoking-related self-identity constructs. *Soc Sci Med* 2023; 320: 115667. 2023/01/16. DOI: 10.1016/j.socscimed.2023.115667.

20. Hizli B. Goal-setting dialogue for physical activity with a virtual coach: code., <https://doi.org/10.5281/zenodo.6647381> (2022).

21. Leipold N, Lurz M, Wintergerst M, et al. Goal-Setting Characteristics of Nutrition-Related mHealth Systems: A Morphological Analysis. In: *ECIS 2022-30TH EUROPEAN CONFERENCE ON INFORMATION SYSTEMS* 2022.

22. Bandura A and Walters RH. *Social learning theory*. Englewood cliffs Prentice Hall, 1977.

23. Adams MA, Sallis JF, Norman GJ, et al. An adaptive physical activity intervention for overweight adults: a randomized controlled trial. *Plos One* 2013; 8: e82901. 2013/12/19. DOI: 10.1371/journal.pone.0082901.

24. Albers N, Neerincx MA and Brinkman W-P. Addressing people's current and future states in a reinforcement learning algorithm for persuading to quit smoking and to be physically active. *Plos One* 2022; 17. DOI: 10.1371/journal.pone.0277295.

25. Albers N, Neerincx MA and Brinkman W-P. Persuading to Prepare for Quitting Smoking with a Virtual Coach: Using States and User Characteristics to Predict Behavior. In: *Proceedings of the 2023 International Conference on Autonomous Agents and Multiagent Systems* 2023, pp.717-726.

26. Albers N. Reinforcement learning-based persuasion for a conversational agent to support behavior change: code., <https://zenodo.org/records/6319356> (2022).

27. van der Burg S, Baccinelli W, Richardson R, et al. PerfectFit (v.1.0.0), <https://doi.org/10.5281/zenodo.10837093> (2024).

28. Versluis A, Penfornis KM, van der Burg S, et al. Targeting key risk factors for cardiovascular disease in at-risk individuals: developing a digital, personalized and real-time intervention to facilitate smoking cessation and physical activity. *Manuscript under review* 2024.

29. Mercken L, Candel M, van Osch L, et al. No smoke without fire: The impact of future friends on adolescent smoking behaviour. *Brit J Health Psych* 2011; 16: 170-188. DOI: 10.1348/135910710x531608.

30. World Health Organization. Physical activity, <https://www.who.int/news-room/fact-sheets/detail/physical-activity> (2022, accessed 17 April 2024).

31. King AC, Pruitt LA, Woo S, et al. Effects of Moderate-Intensity Exercise on Polysomnographic and Subjective Sleep Quality in Older Adults With Mild to Moderate Sleep Complaints. *J Gerontol a-Biol* 2008; 63: 997-1004. DOI: 10.1093/gerona/63.9.997.

32. Prosise GL, Bonnet MH, Berry RB, et al. Effects of Abstinence from Smoking on Sleep and Daytime Sleepiness. *Chest* 1994; 105: 1136-1141. DOI: 10.1378/chest.105.4.1136.

33. Caddick ZA, Gregory K, Arsintescu L, et al. A review of the environmental parameters necessary for an optimal sleep environment. *Build Environ* 2018; 132: 11-20. DOI: 10.1016/j.buildenv.2018.01.020.

34. Bjorngaard JH, Nordestgaard AT, Taylor AE, et al. Heavier smoking increases coffee consumption: findings from a Mendelian randomization analysis. *Int J Epidemiol* 2017; 46: 1958-1967. DOI: 10.1093/ije/dyx147.

35. Hughes JR and Kalman D. Do smokers with alcohol problems have more difficulty quitting? *Drug Alcohol Depen* 2006; 82: 91-102. DOI: 10.1016/j.drugalcdep.2005.08.018.

36. Thomas DT, Erdman KA and Burke LM. Position of the Academy of Nutrition and Dietetics, Dietitians of Canada, and the American College of Sports Medicine: Nutrition and Athletic Performance. *J Acad Nutr Diet* 2016; 116: 501-528. DOI: 10.1016/j.jand.2015.12.006.

37. Limsanon T and Kalayasiri R. Preliminary Effects of Progressive Muscle Relaxation on Cigarette Craving and Withdrawal Symptoms in Experienced Smokers in Acute Cigarette Abstinence: A Randomized Controlled Trial. *Behav Ther* 2015; 46: 166-176. DOI: 10.1016/j.beth.2014.10.002.

38. McCaul KD, Hockemeyer JR, Johnson RJ, et al. Motivation to quit using cigarettes: A review. *Addict Behav* 2006; 31: 42-56. DOI: 10.1016/j.addbeh.2005.04.004.

39. Baillot A, Chenail S, Polita NB, et al. Physical activity motives, barriers, and preferences in people with obesity: A systematic review. *Plos One* 2021; 16. DOI: 10.1371/journal.pone.0253114.

40. Twyman L, Bonevski B, Paul C, et al. Perceived barriers to smoking cessation in selected vulnerable groups: a systematic review of the qualitative and quantitative literature. *Bmj Open* 2014; 4. DOI: 10.1136/bmjopen-2014-006414.

41. Knowles RD. Positive Self-Talk. *American Journal of Nursing* 1981; 81: 535.

42. Husband CJ, Wharf-Higgins J and Rhodes RE. A feasibility randomized trial of an identity-based physical activity intervention among university students. *Health Psychol Behav* 2019; 7: 128-146. DOI: 10.1080/21642850.2019.1600407.

43. Hagger MS. Habit and physical activity: Theoretical advances, practical implications, and agenda for future research. *Psychol Sport Exerc* 2019; 42: 118-129. DOI: 10.1016/j.psychsport.2018.12.007.

44. Seligman MEP, Steen TA, Park N, et al. Positive psychology progress - Empirical validation of interventions. *Am Psychol* 2005; 60: 410-421. DOI: 10.1037/0003-066x.60.5.410.

45. Albers N, Neerincx MA, Aretz NL, et al. Attitudes Toward a Virtual Smoking Cessation Coach: Relationship and Willingness to Continue. *Persuasive Technology*. 2023, pp.265-274.

46. Bickmore T, Schulman D and Yin LX. Maintaining Engagement in Long-Term Interventions with Relational Agents. *Appl Artif Intell* 2010; 24: 648-666. DOI: 10.1080/08839514.2010.492259.

47. Miller WR. Motivational Interviewing with Problem Drinkers. *Behav Psychother* 1983; 11: 147-172. DOI: 10.1017/S0141347300006583.

48. Rollnick S, Butler CC, Kinnersley P, et al. Motivational interviewing. *Bmj-Brit Med J* 2010; 340. DOI: 10.1136/bmj.c1900.

1. <https://github.com/PerfectFit-project/virtual-coach-main> [↑](#footnote-ref-1)
2. [Perfect Fit virtual coaching intervention for smoking cessation and physical activity: Dialogs and exercises](https://osf.io/ngaje/) [↑](#footnote-ref-2)
3. The categorization of the short (optional) activities are based on the results of the following study: Albers, N., & Brinkman, W. (2023). Perfect Fit - Beliefs about and Competencies Built by Preparatory Activities for Quitting Smoking and Becoming More Physically Active. <https://doi.org/10.17605/OSF.IO/CAX6F>. [↑](#footnote-ref-3)
