# Supplementary material for: Protocol of a mixed-methods evaluation of Perfect Fit: A personalized mHealth intervention with a virtual coach to promote smoking cessation and physical activity in adults
Source: Digit Health. 2024 Dec 5;10:20552076241300020. doi: 10.1177/20552076241300020 (PMC11618927; doi:10.1177/20552076241300020)
Supplement: sj-docx-2-dhj-10.1177_20552076241300020 - Supplemental material for Protocol of a mixed-methods evaluation of Perfect Fit: A personalized mHealth intervention with a virtual coach to promote smoking cessation and physical activity in adults [file sj-docx-2-dhj-10.1177_20552076241300020.docx]

**Appendix B**. *Perfect Fit screenshots*

*
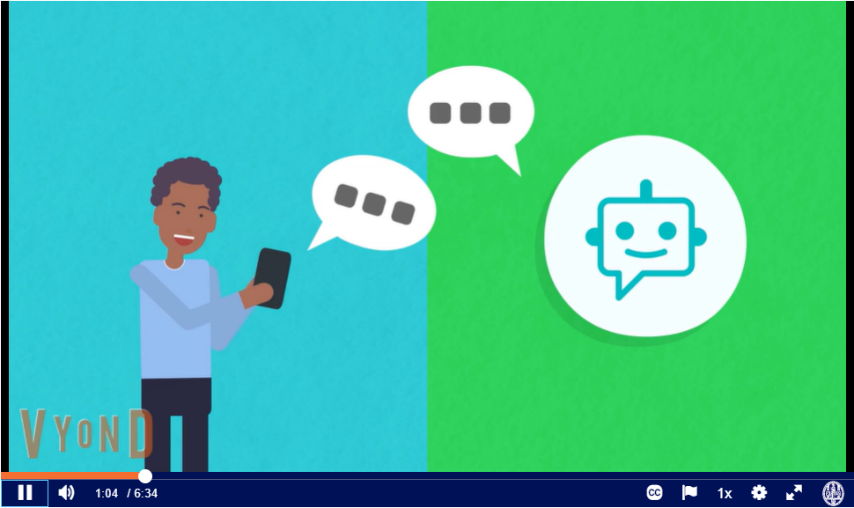
*


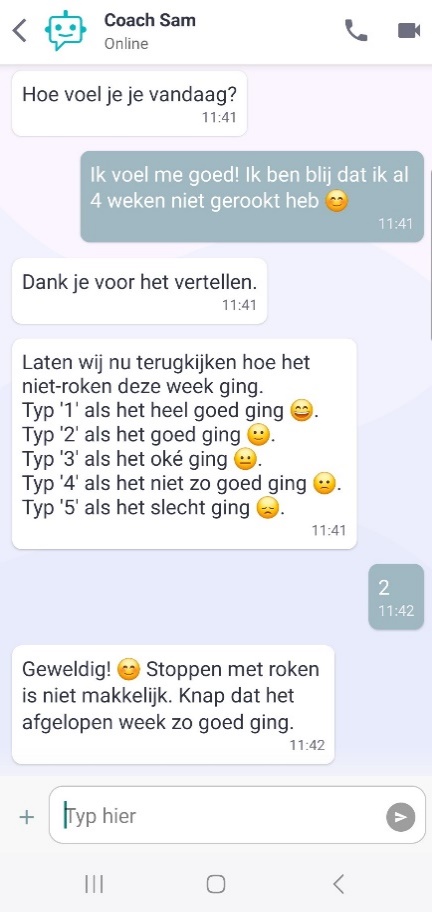
*
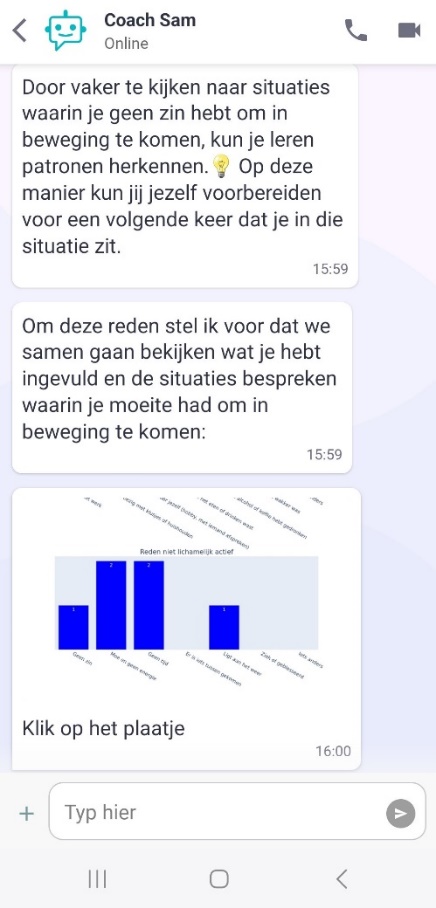

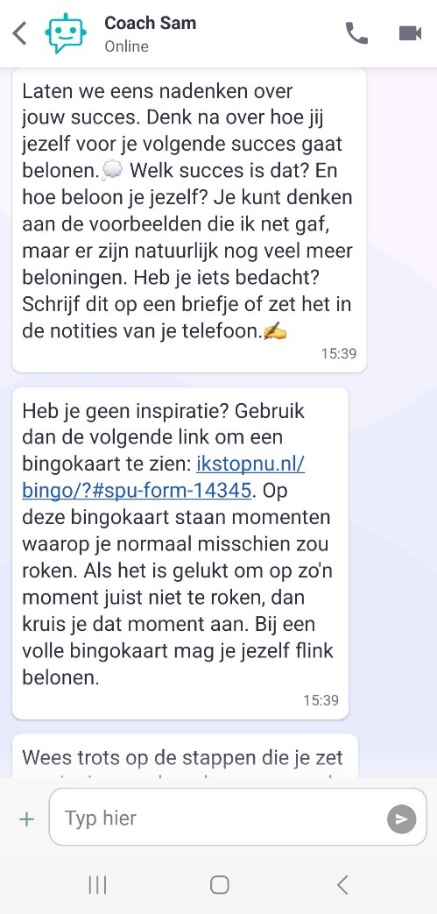
*

**Figure B1.** *Perfect Fit screenshots showing an example of one of the educational videos (top) and three examples of chat dialogs with the virtual coach (in Dutch; lower).*
