# Supplementary material for: Protocol of a mixed-methods evaluation of Perfect Fit: A personalized mHealth intervention with a virtual coach to promote smoking cessation and physical activity in adults
Source: Digit Health. 2024 Dec 5;10:20552076241300020. doi: 10.1177/20552076241300020 (PMC11618927; doi:10.1177/20552076241300020)
Supplement: sj-docx-3-dhj-10.1177_20552076241300020 - Supplemental material for Protocol of a mixed-methods evaluation of Perfect Fit: A personalized mHealth intervention with a virtual coach to promote smoking cessation and physical activity in adults [file sj-docx-3-dhj-10.1177_20552076241300020.docx]

**Appendix C.** *Interview protocol semi-structured individual interviews*

1. **Introduction, explanation of the study**
2. Thank you very much for wanting to participate in this interview. We would like to hear about your experiences with the Perfect Fit program. You may not, or not yet, have completed the program, in which case we will talk about what you thought of it so far. The interview will last approximately 45 to 60 minutes at the most.
3. To thank you for your participation in the interview, you will receive a €25 gift voucher afterward.
4. We would like to formally ask if you consent to participate in the interview. For this, we will start a short recording that will be separate from the interview recording. We are required to save this short recording, which will not include personal information (only audio.) Do you have any questions? Do you agree to start the short, separate, recording to ask your consent to participate in the interview?

***If respondent explicitly agrees:*** *Start recording to obtain informed consent*

***If respondent does not agree:*** *The interview cannot take place*

Are you ready to begin the interview? Then I will now start the recording for the interview... [stop recording of informed consent and start recording of interview]

1. **Interview**
   1. ***Evaluation of behavior change (preliminary effectiveness)***

I would like to ask you a few questions about quitting smoking and increasing your physical activity before we discuss what you thought of the Perfect Fit program.

a. How did it go with quitting smoking (in the last few months of the study)?

*(Always discuss the options below if they are not addressed)*

1. Have you tried to quit smoking? How did that go?
2. Did you smoke in the past week?
3. Have you received smoking cessation support, in addition to the Perfect Fit program?

*If having trouble answering question iii: e.g. visited GP, face-to-face or telephone counseling, medication, nicotine replacement therapy, other smoking cessation app(s)*

b. How did it go with increasing your physical activity?

*(Always discuss the options below if they are not addressed)*

1. Have you tried to increase your physical activity? How did that go? What did you do?
2. Have you received support to increase your physical activity, in addition to the Perfect Fit program?

*If having trouble answering question ii: e.g. face-to-face or telephone counseling, joining a sports association, other physical activity app(s)*

c. You have been working on quitting smoking during the program as well as working on increasing your physical activity. How did you feel about working on both at the same time?

1. What did you like about this?
2. What did you dislike about it?
   1. ***Evaluation of Perfect Fit (feasibility and acceptability of intervention and coach)***

The following questions concern the Perfect Fit program and the support from coach Sam during this program.

1. Did you complete the entire program?
2. How did you use the Perfect Fit program?
3. Can you estimate how long (how many weeks) you used the Perfect Fit program?
4. And how often (e.g., per week) did you use the program?

*If having trouble answering question b: Can you describe a typical week?*

1. Overall, what did you think of the Perfect Fit program?
2. What parts of the Perfect Fit program were you satisfied with? Why?

*Prompts parts: videos, certain conversations with coach Sam, short exercises, tracking steps with the smartwatch, commands*

1. What components were you dissatisfied with? Why?
2. What did you think of ...
3. The short exercises you could do? What did you like, and what could be improved?
4. What did you think of the videos? What did you like, and what could be improved?
5. What did you think of…
6. the preparation phase

*[if needed: The phase in which you set an exercise goal, chose a quit date, and prepared to quit smoking]*

1. the execution phase

*[if needed: The phase that started on your quit date, in which you discussed every week with coach Sam how increasing your physical activity and smoking cessation were going]*

1. the support during difficult moments

*[if needed: In which you could let Sam know using the 'help' command that e.g. you had smoked or found it difficult to be physically active]*

1. closing-dialogue with Sam

*[if needed: The final chat conversation with Sam in which you discussed how it had gone with achieving your goals and made a plan for after the program]*

What did you like, and what could be improved?

1. What did you think of the smartwatch to keep track of your steps? What did you like, and what could be improved?
2. What did you think of Coach Sam?
3. How satisfied were you with coach Sam?
4. Did you feel a connection with coach Sam?
   1. *To what extent did you feel a connection with coach Sam / How would you describe this connection?*
   2. *What parts of the coach made you feel like this? What parts made you feel less like this?*
5. How did you feel about the way coach Sam chatted with you/talked to you? What did you like, and what could be improved?
6. How did you feel about sharing (personal) information with coach Sam?
7. Did you feel you could share your personal information securely in the app?
8. Do you have any other ideas to improve the Perfect Fit program? Which ones?
   1. ***Continuation of Perfect Fit (implementation requirements)***

I would also like to talk to you about the continuation of the Perfect Fit program.

1. You have now used the Perfect Fit program within this study. In what ways do you think the Perfect Fit program should be offered in the future?
2. How would you feel if your primary care physician recommended the Perfect Fit program? Or another health care provider (who do you think is suitable)?
3. How would you feel if your health insurance company offered the Perfect Fit program?
4. How would you feel about searching for the Perfect Fit program yourself in the app store/play store? What would you look for when downloading an app yourself?
5. Do you have any other ideas?
6. What, in your opinion, is important to watch out for as we move forward with the Perfect Fit program?
7. Would you be willing to pay for the Perfect Fit program yourself?
8. If yes, how much?
   1. ***Evaluation of NiceDay (implementation requirements)***
9. What did you think of NiceDay, the app in which the Perfect Fit coach (coach Sam) could be found?
   1. ***Evaluation of the study (study feasibility)***

Finally, I would like to talk to you about your experiences with the study.

1. How did you feel about participating in the study?
2. Do you have any tips for this type of study in the future?
3. **Closing**

Thank you for participating in this interview. Are there any other things you would like to say? *[If time left: What did you personally think was the most important thing we discussed]*
