# Supplementary material for: Protocol of a mixed-methods evaluation of Perfect Fit: A personalized mHealth intervention with a virtual coach to promote smoking cessation and physical activity in adults
Source: Digit Health. 2024 Dec 5;10:20552076241300020. doi: 10.1177/20552076241300020 (PMC11618927; doi:10.1177/20552076241300020)
Supplement: sj-docx-4-dhj-10.1177_20552076241300020 - Supplemental material for Protocol of a mixed-methods evaluation of Perfect Fit: A personalized mHealth intervention with a virtual coach to promote smoking cessation and physical activity in adults [file sj-docx-4-dhj-10.1177_20552076241300020.docx]

**Appendix D.** *Description of primary, secondary and other study outcomes*

1. **Primary outcomes**
   1. ***Intervention’s feasibility***

Feasibility of (the different components of) Perfect Fit will be assessed using *usage data* and a *self-report question on contact frequency with the virtual coach*. Usage data on which components of the intervention (i.e., chat dialogs, informational videos and optional activities) are completed at certain time points will be obtained. Users who complete all the preparation phase dialogs, complete at least one dialog per week during the execution phase of the intervention, and complete the closing dialog, will be marked as adherent users. Usage data will be collected during the intervention (from T0-T1). In addition, at T1 users will be asked to indicate how many times a week they had contact with their virtual coach during Perfect Fit. This self-developed question, which has a free-text response format, complements the usage data.

- 1. ***Intervention’s acceptability***

At T1, participants’ *satisfaction with the Perfect Fit intervention* will be measured with two self-developed Dutch items, e.g. *“Would you use the Perfect Fit program again if needed?”*. The items are scored on 4-point Likert scales with different labels (e.g., from 1 [definitely not] to 4 [yes, definitely]), with higher scores indicating greater satisfaction. Initially, we planned to use the Dutch Client Satisfaction Questionnaire-8^1, 2^ as indicated in the study protocol registration ([NCT06095999](https://clinicaltrials.gov/study/NCT06095999?cond=Perfect%20Fit:%20Evaluation%20of%20a%20Virtual%20Coach%20for%20Smoking%20Cessation%20and%20Physical%20Activity&rank=1)). Instead, we created two self-developed items that better align with the study’s context and use more accessible language, as modifying the original CSQ-8 is not permitted.

- 1. ***Intervention’s usability***

At T1, the *usability of the intervention and the chat function of the NiceDay app* will be measured with a Dutch translation of the System Usability Scale (SUS).^3^ The SUS is a valid and robust tool to determine whether a system, such as an app, is user-friendly.^4^ The total questionnaire consists of ten items with items such as *“I think the virtual coach is unnecessarily complex”* and *“I found the various functions in this system were well integrated”*. The items are rated on a 5-point Likert scale ranging from 1 (strongly disagree) to 5 (strongly agree).

- 1. ***Acceptability and usability virtual coach***

At T1*, acceptance of the virtual coach* will be assessed using a Dutch translation of a set of six items that measure the evaluation of the coach in terms of satisfaction, usability, continuation of collaboration, relationship, preference for a program with or without coach and adherence to advice from the coach. The questionnaire has been used in other studies evaluating virtual coaches.^5, 6^ Two example items are: *“How satisfied were you with the virtual coach?”* and *“How easy was it talking to the virtual coach?”*. Answers are given on a 7-point Likert scale with different labels (e.g., from 1 [not at all satisfied] to 7 [very satisfied]).

- 1. ***Qualitative data on primary outcome measures***

Qualitative individual semi-structured interviews with participants (conducted at T1) will complement the quantitative data and provide more in-depth insight into acceptability, feasibility, preliminary effectiveness, and requirements for implementation of Perfect Fit.

1. **Secondary outcomes**
   1. ***Preliminary effectiveness***
      1. *Self-reported prolonged smoking abstinence after the intervention*

To assess prolonged smoking abstinence at multiple time points after the intervention (T1-T4), a combination of *continuous abstinence* and *7-day point prevalence abstinence* will be used.^7-9^ Participants will be asked if they have smoked since the end of the intervention (yes/no; i.e., continuous abstinence). In case their answer to this question is ‘yes’, participants will be asked if they have smoked the seven days before the assessment (yes/no; i.e., 7-day point prevalence abstinence). In case their answer to this question is ‘no’, two other follow-up questions will be asked, namely: *“How long ago did you smoke your last cigarette, cigar or rolling tobacco?”* and *“How many cigarettes, cigars or rolling tobacco did you smoke?”* Assessing prolonged smoking abstinence, through a combination of continuous and point prevalence abstinence, allows us to assess change over time while taking into account the non-linearity of smoking cessation due to (re)lapses.^8-10^

- - 1. *Self-reported level of physical activity (PA)*

*Self-reported level of PA* will be measured at T0-T4 with a Dutch translation of the 3-item Godin-Shephard Leisure-Time PA questionnaire (GSLTPAQ).^11, 12^ Participants are asked how many times on average, during a typical week, they do strenuous, moderate or mild exercise for more than 15 minutes. Examples of each exercise intensity are provided (e.g., running, soccer, tennis, easy walking). The total score of the questionnaire is calculated by multiplying the number of times per week strenuous exercise by nine, multiplying the number of times per week moderate exercise by five, multiplying the number of times per week mild exercise by three, and taking the sum of this (i.e., [9 x times/wk strenuous] + [5 x times/wk moderate] + [3 x times/wk mild]). A total score of 24 or more is defined as ‘active’, a total score of 14 to 23 is defined as ‘moderately active’ and a score of less than 14 is defined as ‘insufficiently active’.^12^ The questionnaire and this scoring method are shown to be valid to assess PA levels in different adult populations.^e.g., 13^

- - 1. *Non-smoker self-identity*

*Non-smoker self-identity* will be assessed at T0-T4 using seven items,^14^ of which three are adapted from the Abstainer Self-Concept scale and three are adapted from the Smoker Self-Concept Scale^15^ by replacing ‘smoking’ with ‘not smoking’. In addition, the item *“I would like to be a non-smoker”* will be added.^adapted from 16^ All items are translated to Dutch and can be answered on a 5-point Likert scale ranging from 1 (strongly disagree) to 5 (strongly agree). Higher total scores indicate stronger non-smoker self-identity. The Dutch questionnaire, created with the adapted items, has good internal consistency.^14, 17^

- - 1. *PA self-identity*

*PA self-identity* will be measured at T0-T4 with a Dutch translation of the adapted version^18^ of the 9-item Exercise Identity Questionnaire.^19^ PA identity will be measured rather than exercise identity since being physically active entails a broader array of physical activities.^18^ As Perfect Fit aims to enhance PA by increasing the amount of steps, this includes not only sports/exercise, but also daily physical activities like walking to run errands or household chores. The adapted questionnaire shows similar validity characteristics as the original Exercise Identity Questionnaire and has good internal consistency.^18^ Example items of the questionnaire are: *“I consider myself a physically active person”* and *“I need to be physically active to feel good about myself”*. Answers are given on a 7-point Likert scale ranging from 1 (strongly disagree) to 7 (strongly agree) and the total score ranges from 9 to 63, with a higher score indicating stronger identity.

- - 1. *Smoking abstinence self-efficacy*

*Smoking abstinence self-efficacy* will be assessed at T0-T4 with a single item,^adapted from^ ^20^ translated in Dutch. Participants will be asked: *“Overall, how confident are you that you are able not to smoke?”* Similar smoking abstinence self-efficacy questionnaires have been demonstrated to be valid and reliable^e.g., 21, 22^, and abstinence self-efficacy is associated with smoking cessation.^22^ Answers are given on a 5-point Likert scale ranging from 1 (not at all confident) to 5 (extremely confident), with a higher score indicating higher abstinence self-efficacy.

- - 1. *PA self-efficacy*

To assess *self-efficacy for PA* the same Dutch single item^adapted from 20^ will be used for assessing smoking abstinence self-efficacy, but the item will be adjusted to PA behavior. Participants will be asked at T0-T4: *“Overall, how confident are you that you are able to be sufficiently physically active?”* An additional instruction will be provided before the question is asked, explaining the definition of PA (i.e., exercise, but also physical activities like walking to the supermarket) and the recommended levels of PA according to the WHO guidelines.^23^ Similar self-efficacy for PA questionnaires have been demonstrated to be valid and reliable^e.g.,^ ^24^ and PA self-efficacy is associated with actual PA behavior.^25^ Answers are given on a 5-point Likert scale ranging from 1 (not at all confident) to 5 (extremely confident), with a higher score indicating higher self-efficacy for PA.

- - 1. *Objective level of PA: Number of steps (smartwatch)*

The *average number of steps per day* will be measured during the intervention (from T0-T1) using the smartwatches (Garmin Forerunner 55) worn by participants. The step count data recorded by the smartwatches will be transmitted to a sensor data collector app, which will ensure that the data is securely stored at the Perfect Fit server.

- 1. ***Study feasibility***
     1. *Recruitment, response and consent rate*

The feasibility of the study will be assessed in terms of *recruitment, response and consent rate* using participant screening and inclusion logfile data. The following information will be logged: the time between the start of patient recruitment and the day on which the final patient is included (i.e., recruitment rate); proportion of interested patients who completed the screening questionnaire (i.e., response rate); and number of eligible participants who consented to take part in study (i.e., consent rate). Communication with and completed study elements by each participant will be monitored during the full study duration by keeping a participant log file.

- - 1. *Recruitment strategies*

To gain more insight into the *effectiveness of different recruitment strategies*, participants will be asked at T0 how they heard about the study. They can indicate which recruitment strategy was used (e.g., recruited directly by a researcher, via Hartstichting etc.). This will provide insight into effective recruitment strategies for future research, which is especially interesting in the case of participants with a lower socio-economic position as they are often underrepresented in research.

- - 1. *Users’ adherence*

The *adherence of users to the study* *and the intervention* will be assessed using participant log data. Adherence will be assessed in terms of the number of participants who completed the intervention and the entire study, the number of participants who were lost to follow-up over time (dropouts), and the number of participants who remain in the study, i.e. continuing to fill in questionnaires, but no longer actively using the intervention (nonusers).^26^ Adherence will be measured from the completion of the onboarding meeting. Participants who completed informed consent but later decided not to participate before finishing the onboarding meeting will be categorized as individuals who withdrew their informed consent. Adherence will be continuously monitored throughout the study by keeping a participant screening and inclusion logfile. It will also be assessed with the aforementioned usage data and self-report question (see ‘Intervention’s feasibility’).

- - 1. *Intervention completion*

At the start of the T1 questionnaire, users will be asked if they *completed the intervention* (i.e., completed the final closing dialog with the virtual coach) or not (yes/no). In case they have not completed the intervention yet, they are asked in which week of the execution phase of the intervention they are at that moment. This way it can be assessed if participants fill out the T1 questionnaire while having completed the whole intervention or not. It also provides information on how many people complete the intervention in 16 weeks or less.

- - 1. *Qualitative data on secondary outcome measures*

Qualitative individual semi-structured interviews with participants (conducted at T1) will complement the quantitative data and provide more in-depth insight into acceptability, feasibility, preliminary effectiveness, and requirements for implementation of Perfect Fit.

1. **Other variables**
   1. ***Participant characteristics and eHealth literacy***

General information about the participants will be obtained at T0, namely *gender* (i.e., male, female, other), *age* (birth year), *level of education* (i.e., no, primary, secondary, vocational, higher vocational and university education; as a measure for socio-economic position), if they currently have any *physical or psychiatric/mental (chronic) condition(s)* and *eHealth literacy*.

To assess eHealth literacy a validated Dutch translation^27^ of the eHealth Literacy Questionnaire (eHLQ) will be used.^28^ Five of the seven dimensions of the questionnaire will be used: 1) Using technology to process health information; 2) Understanding of health concepts and language; 3) Ability to actively engage with digital services; 4) Feel safe and in control; and 5) Motivated to engage with digital services. Each dimension consists of five items, resulting in 25 items in total. Each item is scored on a 4-point scale (strongly disagree [1], disagree [2], agree [3], strongly agree [4]), with a higher score indicating higher eHealth literacy. A license for the use of the eHLQ was obtained from the copyright holders.

- 1. ***Intention to quit smoking***

At T0, participants will be asked for an *intended timeline to quit smoking*. Participants indicate whether they intend to quit in the future. Answer categories are based on the stages of change of the Transtheoretical model^29^ and include: within the next month (1), between 1 to 6 months from now (2), sometime in the future, beyond 6 months (3) or not planning to quit (4).^30^ Participants who choose answer category four will be categorized as ‘non-intenders’. This variable will be reverse scored so that higher scores indicate a stronger intention to quit. Since the intention to quit smoking is also one of the inclusion criteria, we expect that participants in the study are not likely to choose answer options 3 and 4.

- 1. ***Smoking behavior and physical nicotine dependence***

At T0, *smoking behavior* and *physical nicotine dependence* will be assessed using the Dutch version of the Fagerström Test of Nicotine Dependence (FTND).^31, 32^ The FTND consists of six items, of which four items are scored on a dichotomous scale (yes / no), one item is scored using four answer categories, and one item has an open answer format (asking participants about the number of cigarettes smoked per day). An example item is *“Do you smoke more in the morning than during the rest of the day?”*. Total scores can range from 5 to 12, with a higher score indicating higher nicotine dependence. The FTND is widely used across studies among smokers, with demonstrated reliability.^31, 33^

- 1. ***Intention to become more physically active***

At T0, participants will be asked for an *intended timeline to become more physically active*, using the same question as for intention to quit smoking. First, an instruction will be provided before the question is presented, explaining the definition of PA (i.e., exercise, but also physical activities like walking to the supermarket) and the recommended levels of PA according to the WHO guidelines.^23^ Then participants are asked to indicate whether they intend to become more physically active. Answer categories are based on the stages of change of the Transtheoretical model^29^ and include: within the next month (1), between 1 to 6 months from now (2), sometime in the future, beyond 6 months (3) or not planning to become more physically active (4).^30^ Participants who choose answer category four will be categorized as ‘non-intenders’. This variable will be reverse scored so that higher scores indicate a stronger intention to become more physically active.

- 1. ***Use of additional aids/support for smoking cessation and PA***

Participants will be asked at T1-T4 if they made use of *additional aids or support for smoking cessation and PA*. Participants are asked if they have used any type of additional support (e.g., medication, booklets, websites, other apps, or treatment/support, like professional coaching, a sport challenge or acupuncture) next to Perfect Fit, and if so, what type of support. This will provide insight into other forms of support that were used and could influence the results. It will also provide insight into the applicability of Perfect Fit (e.g., use as stand-alone or add-on intervention).

- 1. ***Self-reported (re)lapses during intervention after quit date***

*Lapses* are defined as one time point of smoking again after the quit date (any quantity) and *relapses* are defined as multiple time points of smoking again after the quit date (any quantity). Usage data about cigarettes smoked after the quit date (i.e., [re]lapses) will be obtained during the execution phase (i.e., the phase that starts on the set quit date) of the intervention. Users can initiate a ‘(re)lapse dialog’ with the virtual coach in case of (re)lapse to receive support from the coach to cope with this. During this dialog, the coach will ask the user if it concerns a lapse or a relapse and how many cigarettes they smoked. Data on (re)lapses rely on the self-report of the user and will be collected from the start of the execution phase to T1.

- 1. ***Smoking e-cigarettes***

At T0-T4, participants will also be asked if they *smoke/use e-cigarettes*. In case their answer to this question is ‘yes’, they will be asked if they smoke e-cigarettes with or without nicotine and how often. This is assessed as some people switch to e-cigarettes after quitting smoking tobacco.

- 1. ***Self-reported level of occupational PA***

Since the level of PA during work can differ between participants, participants will also be asked at T0-T4 about the *level of PA during their workday* using a single self-developed item: *"How would you describe your daily physical activity at work? Choose the answer that best suits your work activities:"*. The answer options are partly based on the Occupational Sitting and PA Questionnaire (OSPAQ)^34^ and the Global PA Questionnaire (GPAQ)^35^ and include: 1) Mostly sedentary (I sit almost all day and do little or no PA), 2) mild exercise (I am involved in light physical activities such as standing and occasionally walking, but not intensively), 3) moderate intensity exercise (I perform activities that require moderate physical exertion, such as fast walking, lifting light loads), 4) strenuous exercise (I mainly engage in physically demanding activities, such as heavy lifting), or 5) I am not working at the moment. Both the OSPAQ^34^ and the GPAQ^35^ are validated questionnaires with good-to-excellent test-retest reliability.

**References**

1. De Wilde EF and Hendriks VM. The client satisfaction questionnaire: Psychometric properties in a dutch addict population. *Eur Addict Res* 2005; 11: 157-162. DOI: 10.1159/000086396.

2. Larsen DL, Attkisson, C. C., Hargreaves, W. A., & Nguyen, T. D. . Assessment of client/patient satisfaction: development of a general scale. *Evaluation and program planning* 1979; 2: 197-207. DOI: 10.1016/0149-7189(79)90094-6.

3. Brooke J. SUS-A quick and dirty usability scale. *Usability evaluation in industry* 1996; 189: 4-7.

4. Bangor A, Kortum PT and Miller JT. An empirical evaluation of the System Usability Scale. *Int J Hum-Comput Int* 2008; 24: 574-594. DOI: 10.1080/10447310802205776.

5. Bickmore TW, Mitchell SE, Jack BW, et al. Response to a relational agent by hospital patients with depressive symptoms. *Interact Comput* 2010; 22: 289-298. DOI: 10.1016/j.intcom.2009.12.001.

6. Provoost S, Kleiboer A, Ornelas J, et al. Improving adherence to an online intervention for low mood with a virtual coach: study protocol of a pilot randomized controlled trial. *Trials* 2020; 21: 1-12. DOI: 10.1186/s13063-020-04777-2.

7. Secades-Villa R, Pericot-Valverde I and Weidberg S. Relative reinforcing efficacy of cigarettes as a predictor of smoking abstinence among treatment-seeking smokers. *Psychopharmacology* 2016; 233: 3103-3112. DOI: 10.1007/s00213-016-4350-6.

8. Velicer WF and Prochaska JO. A comparison of four self-report smoking cessation outcome measures. *Addict Behav* 2004; 29: 51-60. DOI: 10.1016/S0306-4603(03)00084-4.

9. West R, Hajek P, Stead L, et al. Outcome criteria in smoking cessation trials: proposal for a common standard. *Addiction* 2005; 100: 299-303. DOI: 10.1111/j.1360-0443.2004.00995.x.

10. Cheung KL, de Ruijter D, Hiligsmann M, et al. Exploring consensus on how to measure smoking cessation. A Delphi study. *Bmc Public Health* 2017; 17. DOI: 10.1186/s12889-017-4902-7.

11. Amireault S and Godin G. The Godin-Shephard Leisure-Time Physical Activity Questionnaire: Validity Evidence Supporting Its Use for Classifying Healthy Adults into Active and Insufficiently Active Categories. *Percept Motor Skill* 2015; 120: 604-622. DOI: 10.2466/03.27.PMS.120v19x7.

12. Godin G. The Godin-Shephard Leisure-Time Physical Activity Questionnaire. *The Health & Fitness Journal of Canada* 2011; 4: 18-22. DOI: 10.14288/hfjc.v4i1.82.

13. Sikes EM, Richardson EV, Cederberg KJ, et al. Use of the Godin leisure-time exercise questionnaire in multiple sclerosis research: a comprehensive narrative review. *Disability and Rehabilitation* 2019; 41: 1243-1267. DOI: 10.1080/09638288.2018.1424956.

14. Meijer E, Gebhardt WA, Van Laar C, et al. Socio-economic status in relation to smoking: The role of (expected and desired) social support and quitter identity. *Soc Sci Med* 2016; 162: 41-49. DOI: 10.1016/j.socscimed.2016.06.022.

15. Shadel WG and Mermelstein R. Individual differences in self-concept among smokers attempting to quit: Validation and predictive utility of measures of the smoker self-concept and abstainer self-concept. *Ann Behav Med* 1996; 18: 151-156. DOI: 10.1007/Bf02883391.

16. Tombor I, Shahab L, Brown J, et al. Positive smoker identity as a barrier to quitting smoking: Findings from a national survey of smokers in England. *Drug Alcohol Depen* 2013; 133: 740-745. DOI: 10.1016/j.drugalcdep.2013.09.001.

17. Meijer E, Gebhardt WA, van Laar C, et al. Identified or conflicted: a latent class and regression tree analysis explaining how identity constructs cluster within smokers. *BMC Psychol* 2022; 10: 231. 2022/10/08. DOI: 10.1186/s40359-022-00937-y.

18. Strachan SM, Brawley LR, Spink K, et al. Older adults' physically-active identity: Relationships between social cognitions, physical activity and satisfaction with life. *Psychol Sport Exerc* 2010; 11: 114-121. DOI: 10.1016/j.psychsport.2009.09.002.

19. Anderson DF and Cychosz CM. Development of an Exercise Identity Scale. *Percept Motor Skill* 1994; 78: 747-751. DOI: 10.2466/pms.1994.78.3.747.

20. Loprinzi PD, Wolfe CD and Walker JF. Exercise facilitates smoking cessation indirectly via improvements in smoking-specific self-efficacy: Prospective cohort study among a national sample of young smokers. *Prev Med* 2015; 81: 63-66. DOI: 10.1016/j.ypmed.2015.08.011.

21. Etter JF, Bergman MM, Humair JP, et al. Development and validation of a scale measuring self-efficacy of current and former smokers. *Addiction* 2000; 95: 901-913. DOI: 10.1046/j.1360-0443.2000.9569017.x.

22. Spek V, Lemmens F, Chatrou M, et al. Development of a Smoking Abstinence Self-efficacy Questionnaire. *Int J Behav Med* 2013; 20: 444-449. DOI: 10.1007/s12529-012-9229-2.

23. World Health Organization. Physical activity, <https://www.who.int/news-room/fact-sheets/detail/physical-activity> (2022, accessed 17 April 2024).

24. Mendoza-Vasconez AS, Marquez B, Benitez TJ, et al. Psychometrics of the self-efficacy for physical activity scale among a Latina women sample. *Bmc Public Health* 2018; 18. DOI: 10.1186/s12889-018-5998-0.

25. McAuley E and Blissmer B. Self-efficacy determinants and consequences of physical activity. *Exerc Sport Sci Rev* 2000; 28: 85-88. 2000/07/21.

26. Eysenbach G. The Law of Attrition. *J Med Internet Res* 2005; 7. DOI: 10.2196/jmir.7.1.e11.

27. Poot CC, Meijer E, Fokkema M, et al. Translation, cultural adaptation and validity assessment of the Dutch version of the eHealth Literacy Questionnaire: a mixed-method approach. *Bmc Public Health* 2023; 23: 1006. DOI: 10.1186/s12889-023-15869-4.

28. Kayser L, Karnoe A, Furstrand D, et al. A Multidimensional Tool Based on the eHealth Literacy Framework: Development and Initial Validity Testing of the eHealth Literacy Questionnaire (eHLQ). *J Med Internet Res* 2018; 20: e36. Original Paper 12.02.2018. DOI: 10.2196/jmir.8371.

29. Prochaska JO and DiClemente CC. The transtheoretical approach. *Handbook of psychotherapy integration*. 2005, pp.147-171.

30. Hummel K, Candel M, Nagelhout GE, et al. Construct and Predictive Validity of Three Measures of Intention to Quit Smoking: Findings From the International Tobacco Control (ITC) Netherlands Survey. *Nicotine Tob Res* 2018; 20: 1101-1108. 2017/05/05. DOI: 10.1093/ntr/ntx092.

31. Heatherton TF, Kozlowski LT, Frecker RC, et al. The Fagerstrom Test for Nicotine Dependence: A Revision of the Fagerstrom Tolerance Questionnaire. *Brit J Addict* 1991; 86: 1119-1127. DOI: 10.1111/j.1360-0443.1991.tb01879.x.

32. Penfornis KM, Gebhardt WA, Rippe RCA, et al. My future-self has (not) quit smoking: An experimental study into the effect of a future-self intervention on smoking-related self-identity constructs. *Soc Sci Med* 2023; 320: 115667. 2023/01/16. DOI: 10.1016/j.socscimed.2023.115667.

33. Vink JM, Willemsen G, Beem AL, et al. The Fagerstrom Test for Nicotine Dependence in a Dutch sample of daily smokers and ex-smokers. *Addict Behav* 2005; 30: 575-579. DOI: 10.1016/j.addbeh.2004.05.023.

34. Chau JY, Van der Ploeg HP, Dunn S, et al. Validity of the Occupational Sitting and Physical Activity Questionnaire. *Med Sci Sport Exer* 2012; 44: 118-125. DOI: 10.1249/MSS.0b013e3182251060.

35. Armstrong T and Bull F. Development of the World Health Organization Global Physical Activity Questionnaire (GPAQ). *J Public Health-Heid* 2006; 14: 66-70. DOI: 10.1007/s10389-006-0024-x.
